# An evaluation of the quality of self-harm incident reporting across the Australian asylum seeker population according to World Health Organization (WHO) guidelines

**DOI:** 10.1186/s12888-020-02709-7

**Published:** 2020-06-15

**Authors:** Kyli Hedrick, Gregory Armstrong, Guy Coffey, Rohan Borschmann

**Affiliations:** 1 Centre for Mental Health, Melbourne School of Population and Global Health, Faculty of Medicine, Dentistry and Health Sciences, The University of Melbourne, 207 Bouverie Street, Carlton, Victoria 3010 Australia; 2 Nossal Institute for Global Health, Melbourne School of Population and Global Health, The University of Melbourne, 333 Exhibition Street, Melbourne, 3000 Australia; 3 The Victorian Foundation for Survivors of Torture (Foundation House), 4 Gardiner Street, Brunswick, Victoria 3056 Australia; 4 Centre for Health Equity; Melbourne School of Population and Global Health, Faculty of Medicine, Dentistry and Health Science, The University of Melbourne, 207 Bouverie Street, Carlton, Victoria 3010 Australia; 5 Centre for Adolescent Health, Murdoch Children’s Research Institute, 50 Flemington Road, Parkville Victoria, Melbourne, 3052 Australia; 6 Health Service and Population Research Department; Institute of Psychiatry, Psychology & Neuroscience, King’s College London, London, UK; 7 Melbourne School of Psychological Sciences, The University of Melbourne, Melbourne, Australia

## Abstract

**Background:**

Asylum seekers are at elevated risk of self-harm, and the personal and public health costs of self-harm are high; yet the monitoring and reporting of self-harm has been limited and lacking in transparency. This study aims to evaluate the quality of self-harm incident reporting across the Australian asylum seeker population, including by processing arrangements (i.e. community-based, community detention, onshore detention, Nauru, and Manus Island).

**Methods:**

All self-harm incidents reported across the entire Australian asylum seeker population between 1 August 2014 and 31 July 2015 were obtained via the *Freedom of Information Act*. We assessed the quality of self-harm incident reporting according to the World Health Organization (WHO)'s self-harm reporting guidelines.

**Results:**

A total of 949 self-harm incident reports were assessed. Date, location (processing arrangement), and time of self-harm were routinely reported. Gender was recorded in less than two thirds (62.1%) of all incidents. Method(s) used to self-harm was reported in 81.5% of all incidents, though IDC-10 codes were not reported in any episodes. Psychological or psychiatric assessments were recorded after 4.0% of all incidents, most frequently on Manus Island (10.9%), and in Nauru (10.0%), and least frequently in community-based arrangements (1.7%) and in onshore detention (1.4%), and not at all in community detention. Ambulances were reported as attending 2.8% of all episodes. Hospital attendances were reported following 6.0% of all self-harm incidents, with attendances most commonly reported in incidents occurring in community detention (30.3%), and in community-based arrangements (19.4%). Medevac (air ambulances) were recorded as being utilised in 0.4% of all incidents (2.1% of episodes on Nauru, 1.8% on Manus Island).

**Conclusions:**

The findings of our study indicate that the accessibility and quality of self-harm data is substandard and inconsistent with WHO self-harm reporting guidelines. Such variable reporting makes the identification of self-harm trends, the implementation of prevention strategies – including those at a policy level - and the clinical management of self-harm, extremely challenging. Improved self-harm reporting and monitoring is urgently needed for mitigating and responding to self-harm risk among asylum seekers.

## Background

The United Nations High Commissioner for Refugees (UNHCR) reports that the number of displaced individuals worldwide now exceeds 70 million [1]. Since 1992, Australia has had a policy of mandatory immigration detention for all ‘unlawful non-citizens’, the majority of which have been asylum seekers during periods when boat arrivals have been large [2]. This policy has also been extended to offshore processing, most recently reinstated in late 2012. Offshore processing includes the indefinite detention of asylum seekers on the Pacific island nation of Nauru and Manus Island, Papua New Guinea, and precludes their resettlement in Australia [3]. In addition to detaining asylum seekers in both onshore and offshore immigration detention, asylum seekers in Australia may also be held in a form of ‘open’ detention, commonly known as community detention, or allowed to live in the community, under community-based arrangements [4]. Australia’s policy of detaining asylum seekers, and its associated immigration policies, have attracted much national and international attention [5].

There is now a robust body of evidence demonstrating that asylum seekers experience rates of depression, anxiety and post-traumatic stress disorder (PTSD) [6, 7] that are many times higher than in the general community. Studies have also observed that the mental health of asylum seekers worsens with time spent in immigration detention [8]. Whilst research has clearly highlighted the adverse effects of both pre-arrival and post-migration stressors on the mental health of asylum seekers, particularly in relation to immigration detention, research regarding self-harm among asylum seekers has been very limited [9]. Given the available evidence suggests that asylum seekers have many of the known risk factors for self-harm [9, 10] and being detained has been found to further increase self-harm risk [11], such research is urgently needed. Furthermore, the costs of self-harm to individuals, families, and at the public level, are high [12]. Increased knowledge about the epidemiology of self-harm among asylum seekers may help to inform policy and practice development, and the investigation of self-harm among asylum seeker populations should therefore be made a public health priority.

The paucity of research into self-harm in the Australian asylum seeker population can be attributed to the self-harm reporting processes of the Australian government [9, 13]. The government does collect data on all self-harm incidents that are reported as occurring across the Australian asylum seeker population, and some data are made available under certain circumstances, such as parliamentary inquiries [14]. However, it appears that the immigration authorities do not routinely monitor, compile, analyse and report such data, and therefore cannot use these data to improve efforts to prevent and respond to incidents of self-harm. As highlighted in the Commonwealth Immigration Ombudsman’s [14] report into Suicide and Self-harm in the Immigration Detention Network, for example, there were a number of issues with the data supplied by the then-called Department of Immigration and Citizenship (DIAC), making self-harm prevalence rates and contributing factors difficult to ascertain. A number of parliamentary and independent reports [15–17], all corroborate this finding, citing problems with the Department of Immigration’s information management and reporting processes, as well as the lack of independent monitoring of their self-harm reporting practices.

In its 2013 report ‘Preventing suicide: a global imperative’, the World Health Organization (WHO) [18] identified a number of areas for action by governments around the world. One of the main recommendations was improvement in the quality of data on self-harm and suicide attempts by responsible state authorities [18]. To this end the WHO subsequently developed guidelines on establishing and maintaining surveillance systems for self-harm and suicide attempts [19]. These guidelines, contained in a detailed practice manual, can be used by countries wishing to establish, maintain or improve their self-harm reporting and monitoring practices. More specifically, the guidelines highlight the core self-harm data items that countries should collect and routinely report in any self-harm surveillance system. The guidelines [19] assert that improvements in the quality of data can inform the development of the most appropriate setting-specific interventions, as well as help to produce more efficient national self-harm prevention programs.

The primary aim of this study was to assess the quality of self-harm incident reporting across the entire Australian asylum seeker population, according to the core WHO self-harm reporting guidelines [19]. A secondary aim was to determine whether the quality of reporting varied by processing arrangements (i.e. community-based arrangements, community detention, onshore detention, Nauru, and Manus Island).

## Methods

### Dataset: self-harm incident reports

We conducted a content analysis of all self-harm incidents reported as occurring among the Australian asylum seeker population between 1 August 2014 and 31 July 2015. In this context, a self-harm incident is an event involving an asylum seeker in one of Australia’s onshore immigration detention facilities, offshore processing centres, in community detention, or living in community-based arrangements. Self-harm is defined as all forms of intentional self-injury (or self-poisoning), irrespective of suicidal intent or motivation [14]. All self-harm incidents that occur in the Australian asylum seeker population are required to be reported verbally by staff or service providers to a duty manager within 30 minutes, and in writing on an incident report form within three hours [14]. A categorisation (e.g., ‘self-harm’) and the incident details, including a description or summary of the incident, are then entered into the Department of Immigration’s centralised incident management log, where the self-harm data are stored. According to internal reporting guidelines, the self-harm incident reports are supposed to include a description of the method and nature of the injury, the incident’s time and place, and any subsequent action taken [14].

All self-harm incidents were obtained under the *Freedom of Information Act* (FOIA) [20], after being found to meet the public interest test [21] and published on the (then-called) Department of Immigration and Border Protection (DIBP)'s disclosure log [22]. Ethics approval for this study was granted by the University of Melbourne’s Human Research Ethics Committee (#1749949.1).

### Quality assessment

A quality assessment was undertaken in order to evaluate each self-harm incident against the core WHO self-harm reporting guidelines [19]. The coding framework for core self-harm data items outlined in the WHO ‘Practice manual for establishing and maintaining surveillance systems for suicide attempts and self-harm’ [see supplementary file 1] was used to assist the coder in identifying all relevant reporting characteristics. Gender was coded following a qualitative analysis of the text in each report, as the official self-harm incident reports did not include a gender tick box. Where possible, for example, terms such as ‘she’, ‘her’, ‘hers’, ‘female’ and ‘woman’ and ‘he’, ‘him’, ‘his’, ‘male’ and ‘man’ were used to code gender. Unique person number, date of birth, age, and country of origin - an optional WHO self-harm reporting item - were not able to be extracted from the incident reports. Such identifying information, if recorded, would have been redacted prior to release under the FOIA. Details pertaining to country of origin for each processing arrangement were, however, instead retrieved from official DIBP statistics [23, 24], as well as statistics regarding offshore processing sourced by the Refugee Council of Australia [25]. An independent coder was used to assess the quality assessment by examining a sample of 100 incident reports, as a reliability check. The inter-rater reliability was found to be very high (kappa = 0.95) [26] and, on that basis, all remaining events were coded by a single coder (KH). All data were entered into SPSS 24 for descriptive analysis.

## Results

### Descriptive information

A total of 949 self-harm incidents were reported in the Australian asylum seeker population (Table 1). The number of reported self-harm incidents were observed to vary considerably according to processing arrangements (i.e. community-based, community-detention, onshore detention, offshore detention [Nauru], and offshore detention [Manus Island] (Table 2). Whilst country of origin could not be determined from the incident reports, official immigration statistics [23, 24] highlight that the two main source countries of asylum seekers in onshore detention, community detention, as well as community-based arrangements during the study period were Iran and Sri Lanka. The largest numbers of asylum seekers sent to Nauru and Manus island at this time were from Iran, or were stateless [25]. There were also sizeable numbers of asylum seekers sent to Nauru and Manus Island who were from Sri Lanka, Afghanistan, Pakistan and Iraq [25].

**Table 1:** Quality assessment of reporting of all self-harm incidents in the Australian asylum seeker population between 1 August 2014 and 31 July 2015, according to WHO self-harm reporting guidelines

Core WHO self-harm (SH) data items	% (n) (***N*** = 949 unless otherwise specified)
**Basic details of the incident**	
Unique event number	100%
Hospital or medical centre number	0 (0%)
Sex	590 (62.1%)
State/country	100%
Date of SH	100%
Day of week of SH	0 (%)
Time of SH	100%
Primary location of SH incident	100%
**Methods of self-harm**	
Methods of SH	774 (81.5%)
ICD-10 codes	0 (0%)
Multiple methods of SH listed	31 (3.3%)
If intentional self-poisoning (chemicals), name of poison	57/57 (100%)
Quantity of poison	0 (0%)
If intentional self-poisoning (medication), name of medication	34/79 (43.0%)
Quantity of medication	11/79 (13.9%)
Type of foreign object ingested	27/28 (96.4%)
Quantity of foreign object	22/28 (78.5%)
**Medical severity and response**	
Medical severity	64 (6.8%)
Seen by?	232 (24.4%)
History of self-harm (previous self-harm)	0 (0%)
Psychological/psychiatric assessment	34 (4.0%)
Diagnosis (any diagnosis associated with the person)	0 (0%)
Taken to hospital	56 (6.0%)
Ambulance attended	27 (2.8%)
Medevac	4 (0.4)

**Table 2:** Quality assessment of reporting of all self-harm incidents in the Australian asylum seeker population between 1 August 2014 and 31 July 2015, according to WHO self-harm reporting guidelines, by processing arrangements

Core WHO self-harm (SH) data items	Community-based ***n*** = 113 n (%)	Community detention ***n*** = 33 n (%)	Onshore detention ***n*** = 560 n (%)	Nauru ***n*** = 188 n (%)	Manus Island ***n*** = 55 n (%)
**Basic details of the incident**					
Unique event number^a^	113 (100%)	33 (100%)	560 (100%)	188 (100%)	55 (100%)
Hospital or medical centre number	0 (0%)	0 (0%)	0 (0%)	0 (0%)	0 (0%)
Sex	40 (35.3%)	16 (48.4%)	321 (57.3%)	158 (84.0%)	55 (100%)
State/country	113 (100%)	33 (100%)	560 (100%)	188 (100%)	55 (100%)
Date of SH	113 (100%)	33 (100%)	560 (100%)	188 (100%)	55 (100%)
Day of week of SH	0 (0%)	0 (0%)	0 (0%)	0 (0%)	0 (0%)
Time of SH	113 (100%)	33 (100%)	560 (100%)	188 (100%)	55 (100%)
Primary location of SH incident	113 (100%)	33 (100%)	560 (100%)	188 (100%)	55 (100%)
**Methods of self-harm**					
Methods of SH	55 (48.6%)	25 (75.7%)	470 (83.9%)	175 (93.0%)	49 (89.0%)
Methods of SH with ICD-10 codes	0 (0%)	0 (0%)	0 (0%)	0 (0%)	0 (0%)
Multiple methods of SH listed	6 (5.3%)	0 (0%)	15 (2.6%)	10 (5.3%)	0 (0%)
If intentional self-poisoning, name of poison (chemicals)	1/1 (100%)	1/1 (100%)	25/25 (100%)	29/29 (100%)	1/1 (100%)
If intentional self-poisoning, quantity of chemicals	0 (0%)	0 (0%)	0 (0%)	0 (0%)	0 (0%)
If intentional self-poisoning, name of poison (medication)	14/22 (63.6%)	2/4 (50%)	8/39 (20.5%)	8/12 (66.6%)	2/2 (100%)
If intentional self-poisoning, quantity of medication	6/22 (27.2%)	1/4 (25.0%)	4/39 (10.2%)	0 (0%)	0 (0%)
Type of foreign object ingested	–	–	10/10 (100%)	10/10 (100%)	7/8 (87.5%)
Quantity of foreign object ingested	–	–	6/10 (60.0%)	10/10 (100%)	5/8 (62.5%)
**Medical severity and response**					
Medical severity	2 (1.7%)	0 (0%)	9 (1.6%)	43 (22.8%)	10 (18.1%)
Seen by?	28 (24.7%)	11 (33.3%)	30 (5.3%)	125 (66.4%)	38 (69.9%)
History of SH	0 (0%)	0 (0%)	0 (0%)	0 (0%)	0 (0%)
Psychological/psychiatric assessment	2 (1.7%)	0 (0%)	8 (1.4%)	18 (10.0%)	6 (10.9%)
Diagnosis (any diagnosis associated with the person)	0 (0%)	0 (0%)	0 (0%)	0 (0%)	0 (0%)
Taken to hospital	22 (19.4%)	10 (30.3%)	13 (2.3%)	11 (5.8%)	0 (0%)
Ambulance attended	0 (0%)	0 (0%)	0 (0%)	26 (13.8%)	1 (1.8%)
Medevac	0 (0%)	0 (0%)	0 (0%)	4 (2.1%)	1 (1.8%)

^a^ For incidents occurring in onshore detention, community detention, and community-based arrangements, the unique event number was termed ‘incident number’. Incidents occurring in Nauru and Manus Island were given unique Planning and Operational Management System [POMS] ID numbers. POMS was the new centralised database for recording incidents introduced during this period [15]

### Quality assessment of the self-harm incident reporting according to WHO reporting guidelines

Our analysis found that all 949 self-harm incidents were assigned a unique event number (Table 1). The date of self-harm was recorded in all 949 incidents. Day of the week was not recorded for any of the self-harm incidents, although this could be extrapolated from the date of the incident. Information regarding processing arrangements (i.e., community-based arrangements, community detention, onshore detention, offshore detention [Nauru], offshore detention [Manus Island]) was reported for all incidents. Details regarding the type of accommodation for those in onshore detention (i.e. Immigration Detention Centres [IDCs], Immigration Transit Accommodation [ITAs], Immigration Residential Housing [IRH], and Alternative Places of Detention [APODs] [4]) - an optional WHO self-harm reporting item - were reported in 100% of all (560) onshore episodes. The time of self-harm incident was reported in all 949 cases (Table 1).

Gender was reported in 590 (62.1%) of all incidents (Table 1). Whilst gender was able to be identified in 100% of self-harm incidents occurring on Manus Island, this was due to the fact that Manus housed only male asylum seekers and the gender composition of the population could therefore be inferred. Gender was most commonly reported in self-harm incidents in Nauru (84.0%), and least commonly in community-based asylum seekers (35.3%) (Table 2).

Methods of self-harm were reported in 774 (81.5%) self-harm incidents, although WHO International Classification of Diseases Tenth Revision (ICD-10) codes were not reported for any episodes (Table 1). Methods of self-harm were most commonly reported in self-harm incidents occurring on Nauru (93.0%), and most infrequently in self-harm incidents occurring among community-based asylum seekers (48.6%) (Table 2).

For incidents involving self-poisoning by chemicals, the type of chemical used was reported in 100% of episodes, although the quantity of chemicals was not recorded in any cases (Table 1). For incidents involving self-poisoning by medication, the type of medication was reported in 34 (43.0%) episodes, with incidents occurring in Nauru (66.6%), and on Manus Island (100% - although numbers were very small), most frequently recording medication type (Table 2). Quantity of medication was most commonly reported in incidents occurring in community-based arrangements (27.2%), and least commonly in incidents occurring in onshore detention (10.2%).

Medical severity (as defined by the WHO guidelines – see supplementary materials) was reported in 64 (6.8%) of all incidents (Table 1). Medical severity was most commonly reported in self-harm incidents occurring in Nauru (22.8%), followed by those on Manus Island (18.1%) (Table 2). Information about who the individual was seen by following the self-harm incident (e.g., general practitioner, nurse, mental health team, police officer, medical clinic staff member) was reported in 24.4% of all episodes. Psychological or psychiatric assessments were reported as occurring in 4.0% of all self-harm episodes, most frequently on Manus Island (10.9%) and in Nauru (10.0%), and least frequently in community-based arrangements (1.7%) and in onshore detention (1.4%), and not at all in community detention (Table 2). No diagnoses associated with individuals or prior history of self-harm were reported in any incident reports.

Ambulances were reported as attending 2.8% of all self-harm incidents (Table 1). Medevac (medical evacuations or air ambulances) were reported as being utilised in 0.4% of all episodes. Individuals were reported as being taken to hospital in 6.0% of all self-harm incidents. Information about hospital admissions was not reported. Hospital attendances were most commonly reported in incidents among those held in community detention (30.3%), followed by incidents occurring in community-based arrangements (19.4%) (Table 2).

## Discussion

The WHO [18] asserts that surveillance of self-harm is an essential element of national self-harm prevention strategies. It also highlights that improvement in the quality and availability of self-harm data is likely to be needed for all countries [19]. The findings of our study confirm that there are substantial gaps in both the quality and availability of self-harm data across the Australian asylum seeker population, and that there is a very low level of compliance with WHO self-harm reporting guidelines.

### Basic details of the incident

Whilst basic details (incident number, date, location (processing arrangement), and time of self-harm) were routinely reported in all self-harm incidents, the majority of the core WHO self-harm data items were reported in a limited and inconsistent fashion. Reporting was also found to vary considerably according to processing arrangements. It is not known why reporting on gender, for example, was found to be so poor, including across processing arrangements, however the lack of a gender tick box on the self-harm incident reports would have almost certainly made it difficult for such information to be captured efficiently. As women have previously been found to be over-represented in self-harm incidents [27], particularly in detention settings [28], and gender-based differences in the use of method(s) used to self-harm have also been identified [27], improved reporting on gender in self-harm incidents among asylum seekers, including across all sub-populations, is clearly needed to better identify relevant gendered trends in self-harm, as well as prevention measures. Including a gender tick box on all self-harm incident reports would be likely to contribute to a substantial improvement in reporting on gender in future.

### Method(s) used to self-harm

According to the WHO [19], the classification of self-harm according to IDC-10 codes ensures that the data are more easily transferrable and can be used to inform national and international self-harm surveillance strategies. The fact that IDC-10 codes were not recorded in any self-harm incidents in our study may therefore have impeded any attempts to make potentially useful comparisons with other national and international populations of relevance, for example, prison populations, as well as the general Australian community. Furthermore, method(s) used to self-harm should have been reported for each self-harm incident, in order to better respond to risk, including through WHO recommended prevention strategies [19], such as means reduction. Without the tracking of trends in method(s) used to self-harm, such strategies clearly cannot be implemented.

Method(s) used to self-harm were also found to be reported inconsistently across the various processing arrangements. The frequency of reporting of method(s) used to self-harm among community-based asylum seekers – the lowest observed across all Australian asylum seeker populations - is likely to be reflective of the type of contact that asylum seekers under such arrangements have with their appointed case workers [29]. That is to say, due to their infrequent contact with their clients, case workers may not have been made aware of the method(s) used, or have inquired about the methods(s) used, and therefore were not able to report such information. It is also possible that case workers may have been notified that a self-harm incident had occurred via a third party (e.g., a health professional, or hospital social worker), but that they were not privy to the confidential details regarding the incident itself.

The quality of reporting in regards to self-poisoning (by both medication and chemicals) in the present study was also found to be particularly poor. Such variable reporting across the entire population makes the identification of self-harm trends, the implementation of means reduction strategies, and the clinical management of self-harm, extremely challenging. Improved reporting of method(s) used to self-harm across the asylum seeker population, including by sub-populations, as well as details regarding the name and quantity of medication and/or poison ingested, is urgently needed for mitigating and responding to future self-harm risk among asylum seekers.

### Medical severity and response

Whilst the Royal Australian and New Zealand College of Psychiatrists (RANZCP) practice guidelines [30] espouse that 100% of individuals who require or present for treatment following an episode of self-harm should undergo psychosocial assessments, such assessments were reported in just 4.0% of all self-harm incidents in the current study. Previous research regarding hospital-treated self-harm has also observed low levels of compliance with similar (United Kingdom) recommendations regarding psychosocial assessments [31, 32], however assessments occurred in 30–50% of episodes, depending on time of day. This represents a rate 7 to 12-fold higher than that reported in the present study. It is possible that psychosocial assessments did occur in relation to the self-harm incidents we reviewed, but were simply not reported, or that they were conducted, but outside the 3-hour reporting period. Regardless of whether or not this is the case, these reporting practices can still be deemed highly problematic as they would have impeded the systematic identification of self-harm trends and prevention measures. Indeed, research regarding hospital-treated self-harm has found that repetition of self-harm is higher among individuals who have not received a psychosocial assessment compared with those who have [33]. Consequently, it may be that the practice of not providing a psychosocial assessment may have exacerbated, rather than reduced, levels of psychosocial distress.

The pattern of reporting observed in regard to hospital attendances – most common in community-based asylum seekers - may reflect the availability and closer proximity of hospitals in mainland Australia. It may also reflect the fact that there are some on-site medical facilities in onshore and offshore immigration detention, rather than the lack of need for hospital treatment in these settings [3, 34, 35]. Access to appropriate medical treatment has previously been found to be particularly lacking in offshore detention [3, 34, 35]. Indeed, medevac (or air ambulances) were only utilised in incidents occurring in Nauru and on Manus Island, which is conceivably due to the remote geographical location of both islands, as well as the reduced capacity of services to respond to particular medical needs and/or emergencies in these settings [3, 34, 35].

The inconsistencies in reporting observed across all processing arrangements likely highlight the fact that a variety of different service providers and government contractors are engaged to provide healthcare, welfare, support and security services across the Australian asylum seeker population, resulting in variable levels of service, as well as reporting and other practices. Indeed, as outlined in the Auditor General’s report into garrison and welfare support [16], as well as the Australian Lawyer’s Alliance report [36] regarding general incident reporting during a similar period, there were a number of inconsistencies in reporting practices and processes observed across different processing arrangements. These were found to arise due to a lack of appropriate information management processes, the ability to engage and retain staff (particularly in offshore detention), and ad-hoc responses to operational events [16]. Perhaps most critically, such inconsistencies were also attributed to differing understandings of what constitutes a notifiable incident, as well as the relationship between incident reporting and risk mitigation. Self-harm (and other) incident reporting and management should be standardised, as recommended by the WHO [19], with formal training provided at regular intervals across all processing arrangements. The findings of the current study call into question the level of information and training provided to staff and contractors regarding incident reporting, as well as responding to, and mitigating, risk.

### Practice implications

Our findings point to the urgent need to institute independent monitoring and surveillance of self-harm in the Australian asylum seeker population. Such monitoring should be transparent and conducted by an independent body of clinical experts who have the statutory power to investigate self-harm among asylum seekers and refugees in both onshore and offshore immigration detention arrangements, as well as in community-based settings. The Australian Medical Association (AMA) has long advocated for the creation of a national statutory body of clinical experts with the power to investigate the health and wellbeing of asylum seekers and refugees [37]. The independent monitoring of self-harm in asylum seekers could potentially form part of the responsibilities of an authority of this kind, with reporting conducted in line with WHO self-harm guidelines [19].

Regular data analysis, tracking and formal reporting of trends in self-harm should also occur, in order to help identify prevention measures, and to assist with clinical management and means reduction strategies. In keeping with the WHO self-harm guidelines [19], the annual incidence rate per 1000 asylum seekers (rather than per 100,000, given the size of the population/s in question) should be calculated for the total Australian asylum seeker population, and disaggregated by gender, processing arrangements, and other subpopulations, based on average annual adult population figures. These population figures can be extracted from statistics the Department of Home Affairs regularly compiles on the number of asylum seekers in both onshore and offshore detention, as well as in community detention, and community-based arrangements [23, 24]. Individual rates of self-harm should be calculated in addition to episode rates of self-harm - as well as 95% confidence intervals (CI) for each of the rates - in order to distinguish between the number of individuals self-harming and the total number of self-harm incidents. As per the WHO guidelines [19], the analysis and reporting of self-harm rates, trends (such as in method(s) used to self-harm), and relevant comparisons (e.g., by gender, or sub-populations) should occur on a quarterly basis. Such reporting could then be used to inform the Commonwealth Government, the UNHCR, the Australian Human Rights Commission, relevant health bodies (e.g., RANZCP), as well as other agencies, such as the private contractors and service providers, (e.g., International Health and Medical Services), which manage or work within the immigration detention network.

In addition to the independent monitoring and reporting of self-harm, a mechanism to ensure that psychosocial assessments are provided for all asylum seekers following every self-harm incident should be established. The regular monitoring of such assessments, as well as any follow-up, could also be conducted by an independent statutory body of clinical experts. Such a mechanism may assist in reducing both self-harm repetition [33], and suicide risk [32], as found in previous research.

The inconsistencies in the quality and breadth of reporting found between all processing arrangements in the present study also highlight the need for standardised training in self-harm reporting for staff and contractors to be implemented across the Australian asylum seeker system. Such training should be provided at regular intervals and include a component on the relationship between incident reporting and risk mitigation. It should also be provided by an independent body. This will help to ensure that a standardised approach to, and understanding of, self-harm incident reporting and risk mitigation is established across the whole asylum seeker population.

### Strengths and limitations

Our study had a number of strengths. First, these are the first published data examining the self-harm reporting practices across the Australian asylum seeker population according to the core WHO self-harm reporting guidelines. Second, we were able to access - via the FOIA – all self-harm incidents reported across the Australian asylum seeker population over the 1 year study period. Finally, our sample was large and enabled us to assess not only the quality of self-harm reporting across the Australian asylum seeker population as a whole, but also the quality of reporting practices according to each of the five main processing arrangements (i.e., community-based asylum arrangements, community detention, onshore detention, offshore detention [Nauru], and offshore detention [Manus Island]).

Our study also had some limitations. The number of unreported episodes of self-harm over the course of the study is not known. As most self-harm is not followed by help-seeking behaviour [38], it is highly likely that the number of self-harm episodes reported represents an under-estimate of the true incidence of self-harm occurring across the Australian asylum seeker population. Furthermore, inquiries and independent reports into conditions and practices in immigration detention have highlighted a number of inconsistencies with incident reporting practices, including cases where incident reports have been destroyed [15, 16, 36]. The current study sought to assess the quality of self-harm reporting according to the core WHO self-harm reporting guidelines [19]. Only data items that were reported as occurring could be assessed. It is possible, therefore, that whilst particular services or responses, for example, psychosocial assessments, or ambulance attendances, were not reported, they did actually occur. It is also possible that additional core self-harm data may have been contained in the hard copies of the self-harm incident reports that the service providers are initially required to complete, prior to entering the incident details directly into the Department of Immigration’s centralised incident management system. As reported by the Department of Immigration on their FOI disclosure log in response to requests for such data [22], however, these incident reports fail to meet the ‘public interest test’ [21] - which would permit their release under the FOIA - because the documents contain ‘substantially the same subject matter’ [22] to that entered and held on the Department of Immigration’s centralised incident management log, and from which it draws its official self-harm data. An inspection and comparison of 20 hard copies of the incident reports (released by a ‘whistle-blower’) with the relevant reports from the study period entered into the Department of Immigration’s critical incident data management system by the authors confirms this: no additional core WHO self-harm data were able to be extracted from such reports. Finally, as the self-harm incidents occurred in 2014–2015, the age of these data could also potentially be considered a limitation. This was, however, the largest set of self-harm data from across the entire Australian asylum seeker population ever made publicly accessible. Furthermore, as no additional self-harm data has been freely released by the government or under FOIA laws in the intervening period, these data remain the largest, and the only currently accessible self-harm dataset from across the entire Australian asylum population.

## Conclusions

The findings of our study indicate that the accessibility and quality of self-harm data is substandard and inconsistent with WHO self-harm reporting guidelines. They also highlight a number of inconsistencies in the quality and breadth of self-harm reporting between all processing arrangements. Such variable reporting across the entire asylum seeker population makes the identification of self-harm trends, the implementation of prevention strategies – including those at a policy level - as well as the clinical management of self-harm, extremely difficult. Improved reporting is urgently needed in order to mitigate and respond to self-harm risk in the Australian asylum seeker population. Regular monitoring, as well as standardised training, should be implemented as an urgent public health priority. The monitoring of self-harm should be conducted by an independent body of clinical experts who have the statutory power to investigate self-harm among asylum seekers in both detention and community-based settings.

## Supplementary information

**Additional file 1.**

## Data Availability

The dataset that support the current findings was generated by data that are publicly available on the Department of Home Affairs’ Freedom of Information Disclosure log at: https://www.homeaffairs.gov.au/access-and-accountability/freedom-of-information/disclosure-logs.

## Abbreviations

AMA: Australian Medical Association
APODs: Alternative Places of Detention
CI: Confidence Intervals
DIAC: Department of Immigration and Citizenship
DIBP: Department of Immigration and Border Protection
FOI/A: Freedom of Information Act
ICD-10: WHO International Classification of Diseases Tenth Revision
IDC: Immigration Detention Centre
IRH: Immigration Residential Housing
ITA: Immigration Transit Accommodation
Medevac: Medical evacuation (or air ambulance)
PTSD: Post-traumatic Stress Disorder
RANZCP: Royal Australian and New Zealand College of Psychiatrists
SH: Self-harm
UNHCR: United Nations High Commissioner for Refugees
WHO: World Health Organization
