# Supplementary material for: An evaluation of the quality of self-harm incident reporting across the Australian asylum seeker population according to World Health Organization (WHO) guidelines
Source: BMC Psychiatry. 2020 Jun 15;20:301. doi: 10.1186/s12888-020-02709-7 (PMC7296646; doi:10.1186/s12888-020-02709-7)
Supplement: Supplementary file 1 — Additional file 1. [file 12888_2020_2709_MOESM1_ESM.pdf]

Practice manual for establishing and maintaining surveillance systems for suicide attempts and self-harm

World Health Organization

WHO Library Cataloguing-in-Publication Data

Practice manual for establishing and maintaining surveillance systems for suicide attempts and self-harm.

1.Suicide, Attempted. 2.Suicide – prevention and control. 3.Suicide – statistics and numerical data. 4.Suicidal Ideation. 5.Self-Injurious Behavior - epidemiology. 6.Registries. 7.Incidence. 8.Public Health Surveillance. 9.Handbooks. I.World Health Organization

ISBN 978 92 4 154957 8
(NLM classification: HV 6545)

© World Health Organization 2016

All rights reserved. Publications of the World Health Organization are available on the WHO website (http://www.who.int) or can be purchased from WHO Press, World Health Organization, 20 Avenue Appia, 1211 Geneva 27, Switzerland (tel.: +41 22 791 3264; fax: +41 22 791 4857; email: bookorders@who.int).

Requests for permission to reproduce or translate WHO publications –whether for sale or for non-commercial distribution– should be addressed to WHO Press through the WHO website (http://www.who.int/about/licensing/copyright_form/index.html).

The designations employed and the presentation of the material in this publication do not imply the expression of any opinion whatsoever on the part of the World Health Organization concerning the legal status of any country, territory, city or area or of its authorities, or concerning the delimitation of its frontiers or boundaries. Dotted and dashed lines on maps represent approximate border lines for which there may not yet be full agreement.

The mention of specific companies or of certain manufacturers' products does not imply that they are endorsed or recommended by the World Health Organization in preference to others of a similar nature that are not mentioned. Errors and omissions excepted, the names of proprietary products are distinguished by initial capital letters.

All reasonable precautions have been taken by the World Health Organization to verify the information contained in this publication. However, the published material is being distributed without warranty of any kind, either expressed or implied. The responsibility for the interpretation and use of the material lies with the reader. In no event shall the World Health Organization be liable for damages arising from its use.

Printed by the WHO Document Production Services, Geneva, Switzerland

Contents

03 Foreword

04 Acknowledgements

05 **01 Introduction**
05 1.1 Background
06 1.2 The need for a manual for establishing and maintaining surveillance systems for suicide attempts and self-harm
08 1.3 Benefits of surveillance systems for hospital-presented suicide attempts and self-harm
10 1.4 Aim of this manual
11 1.5 Who this manual is for
11 1.6 Contents of the manual
11 1.7 References

13 **02 Development and implementation of a surveillance system on suicide attempts and self-harm**
15 2.1 Informing and engaging governments and relevant stakeholders (STEP 1)
16 2.2 Setting up the surveillance system (STEP 2)
16 2.2.1 Costs and potential funding sources
17 2.2.2 Appoint key function staff
17 2.2.3 Standard operating procedures
22 2.3 Ethical requirements, confidentiality and data protection (STEP 3)
22 2.4 Implementation (STEP 4)
23 2.4.1 Enhance and refine standard operating procedures
23 2.4.2 Develop a data registration form
24 2.4.3 Coding and data entry
25 2.4.4 Data compilation
26 2.4.5 Data cleaning
26 2.4.6 Data analysis
27 2.4.7 Data interpretation
30 2.5 References

31 **03 Training of staff involved in data collection**
31 3.1 Definitions
32 3.2 Exclusion and inclusion criteria
32 3.2.1 Exclusion criteria
33 3.2.2 Inclusion criteria
35 3.3 Exclude or include?
41 3.4 Vignettes for practice
49 3.5 References

50 **04 Reporting of surveillance outcomes and dissemination**
50 4.1 Annual reports (STEP 5)
53 4.2 Evidence briefs (STEP 5)
53 4.3 Papers for peer reviewed journals (STEP 5)
54 4.4 Informing policy (STEP 5)
54 4.5 References

55 **05 Maintenance and sustainability over time**
55 5.1 Maintaining standardization and quality of data collection (STEP 5)
56 5.2 Sustainability over time (STEP 5)

57 **06 Terminology**
57 6.1 Introduction
57 6.2 A brief history of terms
61 6.3 References

62 **07 Overview of existing surveillance systems or projects for suicide attempts and self-harm**
62 7.1 Background
63 7.2 Surveillance of suicide attempts and self-harm across the world
64 7.2.1 Dedicated registries for hospital-presented suicide attempts and self-harm
65 7.2.2 Multicentre projects
66 7.2.3 National/international statistics and databases
67 7.2.4 Individual studies
67 7.3 References

68 **08 Relevant reading**

69 Annex 1. Example of an information leaflet describing a surveillance system for suicide attempts and self-harm
70 Annex 2. Example of a paper data registration form
71 Annex 3. Examples of methods used in surveillance of hospital-presented suicide attempts and self-harm

Foreword

Suicide is a complex phenomenon. It is estimated that over 800 000 people die by suicide each year, with many more attempts for each death that occurs. Importantly, suicides are preventable. Timely and effective evidence-based interventions play a key role in preventing suicides and, in order to determine what is effective, good data are needed as a priority. Governments are in a unique position to develop and strengthen surveillance and to provide and disseminate data that can help to inform action.

In its report *Preventing suicide: a global imperative*, WHO set out a number of areas for action for governments, regardless of their level of progress in implementing suicide prevention activities. A key area was the improvement of data on suicides and suicide attempts. This manual builds on these areas of focus by providing practical steps and guidance on setting up a surveillance system for suicide attempts and self-harm within countries. Efforts have been made to improve the registration of suicides; however, a renewed emphasis on the registration of suicide attempts, and self-harm in particular, can add valuable information to guide the design of suicide prevention strategies, as previous suicide attempts are an important risk factor for future attempts and for death by suicide. The manual includes consideration of stakeholder engagement, funding and staffing, through to data collection, collation and analysis.

This is a practical manual that will allow policy-makers to prioritize and guide the implementation of a surveillance system for suicide attempts and self-harm in their respective countries. Users of the manual are encouraged to adapt the steps realistically to match the resources available in their specific context and to ensure sustainability. Importantly, improving the quality of data can help to guide and prioritize the best interventions in each context and contribute to an effective overall suicide prevention strategy.

Dr Shekhar Saxena
Director
Department of Mental Health and Substance Abuse
World Health Organization

Acknowledgements

SUPERVISION AND GUIDANCE

Shekhar Saxena (WHO)

PROJECT COORDINATION AND EDITING

Alexandra Fleischmann (WHO), Sutapa Howlader (Australia)

TECHNICAL CONTRIBUTION AND DRAFTING

Ella Arensman, Eileen Williamson, Grace O'Regan, Justina Hurley, and Eve Griffin (all from the National Suicide Research Foundation and Department of Epidemiology and Public Health, University College Cork, Ireland)

REVIEW

WORKSHOP PARTICIPANTS

Nor Hayati Ali (Malaysia), Chantale Buerli (Switzerland), Fatma Charfi (Tunisia), Eleanor Sonia Davis (Belize), Diego De Leo (Australia), Annette Erlangsen (Denmark), Richardo Gabriel Goti Valdés (Panama), Suzana A. Guerrero Martinez (Dominican Republic), Gopalkrishna Gururaj (India), Ahmad Hajebi (Iran), Guo-Xin Jiang (Sweden), Kairi Kõlves (Australia), Xianyun Li (China), Seyed Kazem Malakouti (Iran), Chihiro Matsumato (Japan), Dinah P. Nadera (Philippines), Lkhagvasuren Nasantsengel (Mongolia), Pia Oetiker (Switzerland), Jong-Ik Park (Republic of Korea), Michael Schoenbaum (USA), Afef Skhiri (Tunisia), Marike Solvalu (Fiji), Tadashi Takeshima (Japan), Rosa Maria Vargas Alvarado (Costa Rica), Yeshi Wangdi (Bhutan)

INTERNATIONAL EXPERTS

Annette Beautrais (New Zealand), José Manoel Bertolote (Brazil), Cindy Claassen (USA), Aislinne Freeman (Ireland), Stephanie Machel (Canada), Edward Mantler (Canada), Lars Mehlum (Norway), Mark Olfson (USA), Jane Pearson (USA), Michael R Phillips (China), Beverly Pringle (USA), Morton Silverman (USA), Gregory Simon (USA), Jean-Pierre Soubrier (France), Marjolijn van den Berg (Belgium)

WHO HEADQUARTERS AND REGIONAL OFFICES

Elisabet Arribas-Ibar, Kidist Bartolomeos, Dan Chisholm, Khalid Saeed, Xiangdong Wang

INTERN

Lakshmi Chandrasekaran

ADMINISTRATIVE SUPPORT

Adeline Loo (WHO)

PRODUCTION

Editing: David Bramley, Switzerland
Graphic design and layout: Yusuke Nakazawa, Japan

WHO would like to thank the Government of Japan and the Government of the Republic of Korea for their financial contributions; and Syngenta, Switzerland for its contribution to the printing of this manual.

01

Introduction

Section overview

Background to surveillance:
The importance of a surveillance system for suicide attempts and self-harm.

Sources of data for surveillance:
Data sourced from medical records in hospitals, usually the emergency department or trauma unit depending on facilities in the country concerned.

The aim of the manual and the benefits of surveillance:
To assess the extent of the problem of hospital presentations due to suicide attempts and self-harm to inform public health policies on prevention, intervention and treatment.

Who the manual is for:
Health professionals, data registration officers, researchers and statisticians working at general hospitals, university departments and research institutes, ministries of health, nongovernmental organizations.

1.1 Background

Public health surveillance is essential to the practice of public health, to guide prevention, monitor activities and evaluate outcomes of such activities (1). The WHO report *Preventing suicide: a global imperative* emphasizes surveillance of suicide and suicide attempts as a core component of national suicide prevention strategies (2). That report followed the WHO Mental Health Action Plan, 2013–2020, by which all WHO Member States committed themselves to work towards the global target of reducing the suicide rate in countries by 10% by 2020 (3).

In terms of practice manuals, WHO has previously published other manuals – such as Injury surveillance guidelines (4), Guidelines for conducting community surveys on injuries and violence (5) and Fatal injury surveillance in mortuaries and hospitals: a manual for practitioners (6). It is important to highlight that improvement in the availability and quality of data on both suicide mortality and suicide attempts is needed for all countries, and that there are major differences between countries in procedures for recording suicide (7, 2).

WHO's 2014 global report on preventing suicide identified a need for guidance on the surveillance of suicide attempts presenting to general hospitals. Currently, the number of countries that have established a surveillance system for suicide attempts is limited, and comparison between established systems is often hindered by differences between systems (2). This manual aims to provide a tool for countries to use in setting up a public health surveillance system for suicide attempts and self-harm cases presenting to general hospitals, based on medical records.

Improved surveillance and monitoring of suicide attempts and self-harm is a core element of the public health model of suicide prevention (8, 9, 2).

When a person presents at a hospital, often in an emergency situation, it should be possible for a clinician to state in the medical records whether or not the injury or poisoning was self-inflicted and whether the injury or poisoning was intentional or accidental. However, the intention to die can be more difficult to ascertain (and therefore to record) since in certain cases even the individual involved may not be certain about his or her intentions. This is why a hospital-focused surveillance system will inevitably represent cases of intentional self-harm with varying levels of suicidal intent and varying underlying motives, and not only suicide attempts characterized by high levels of suicidal intent. For reasons of simplicity of language, the term “suicide attempt” may be used interchangeably with “self-harm” in this document when referring to surveillance systems.

1.2 The need for a manual for establishing and maintaining surveillance systems for suicide attempts and self-harm

It is estimated that, for each suicide, there are likely to have been more than 20 suicide attempts (2). Having engaged in one or more acts of attempted suicide or self-harm is the single most important predictor of death by suicide (10, 11, 12, 13, 14). Consequently, long-term monitoring of the incidence, demographic patterns and methods involved in cases of attempted suicide and self-harm presenting at hospitals in a country or region provides important information that can assist in the development of suicide prevention strategies. By combining this with information on suicide deaths, case fatality rates can be estimated which will assist in identifying high-risk individuals (12, 2).

Based on international research, Figure 1.1 gives a visual representation of the extent of suicidal behaviours. The extent to which cases become known is often compared to an iceberg, where only the tip is visible (suicide, suicide attempts and self-harm presenting to hospitals, and suicide attempts and self-harm presenting to primary care services) while the majority of suicide attempts remain “hidden” under the surface and remain unknown to health services (15, 16, 17).

There are two primary methods for obtaining information about suicide attempts: self-reports of suicide attempts in surveys of representative samples of the population, and medical records that catalogue suicide attempts presenting to health-care facilities (usually hospitals) (2).

This manual focuses on the use of medical records from hospital data as the basis for establishing a surveillance system of suicide attempts and self-harm (Figure 1.1).

When the WHO International Classification of Diseases Tenth Revision (ICD-10) is used, the coding is the same both nationally and internationally and thus data obtained are more likely to be transferable and useful for informing both national and international surveillance programmes. Classification of both suicide and intentional self-harm according to ICD-10 ensures uniformity and consistency of recorded information at global level.

Dedicated hospital-based surveillance systems of suicide attempts and self-harm range from national registries, such as in Ireland (18, 19), to subnational registries at the regional level, such as in the United Kingdom of Great Britain and Northern Ireland, where in Derby, Manchester and Oxford, data are collected on suicide attempts and self-harm presenting to hospital emergency departments (20). In most low- and middle-income countries there are no hospital-based surveillance systems for suicide attempts. However, in recent years, a growing number of low- and middle-income countries have taken first steps towards setting up local registries of suicide attempts presenting to one or more hospitals, such as those in India (21) and Jamaica (22). Data from these individual registries are proving to be very informative and demonstrate the value of having such systems in place in low- and middle-income countries where rates of attempted suicide and self-harm are high and where patient profiles, particularly sex and age, can be quite different from those of high-income countries (2).

In countries with large populations, where national dedicated registries would be difficult to manage, data on suicide attempts and self-harm can sometimes be obtained from national health information systems which are based on various representative data collection approaches. In the United States of America, for example, information on non-fatal suicidal behaviour is collated by the Web-based Injury Statistics Query and Reporting System (WISQARS) and comes from a variety of data sources, including the National Electronic Injury Surveillance System – All Injury Program (NEISS-AIP), the National Trauma Databank (NTDB) of the American College of Surgeons, and the National Poison Data System (NPDS).

Other examples include Canada – where the National Trauma Registry collected information on intentional injuries and where the Ontario Trauma Registry continues to gather data on intentional and unintentional injuries from 11 leading hospitals (23) – as well as Australia, where a State Trauma Registry is in operation in Victoria, providing information on intentional injuries (24).

The lack of internationally standardized methods for the collection of data relating to suicide attempts and self-harm has led to methodological differences in data collection and surveillance. A more uniform approach to the surveillance of hospital-presented suicide attempts and self-harm will foster comparability and understanding of the global picture of suicide attempts and self-harm.

Another element to consider is the legal status of suicide and its impact on the reporting of suicide and suicide attempts. When suicide is considered a criminal act in a country, suicide attempts are often concealed and suicide may remain unreported, which may lead to an incomplete picture (2). A similar obstacle to arriving at a complete picture may stem from insurance policies that do not cover suicide attempts and suicides, and hence these are not reported.

Figure 1.1
The extent of suicidal behaviour

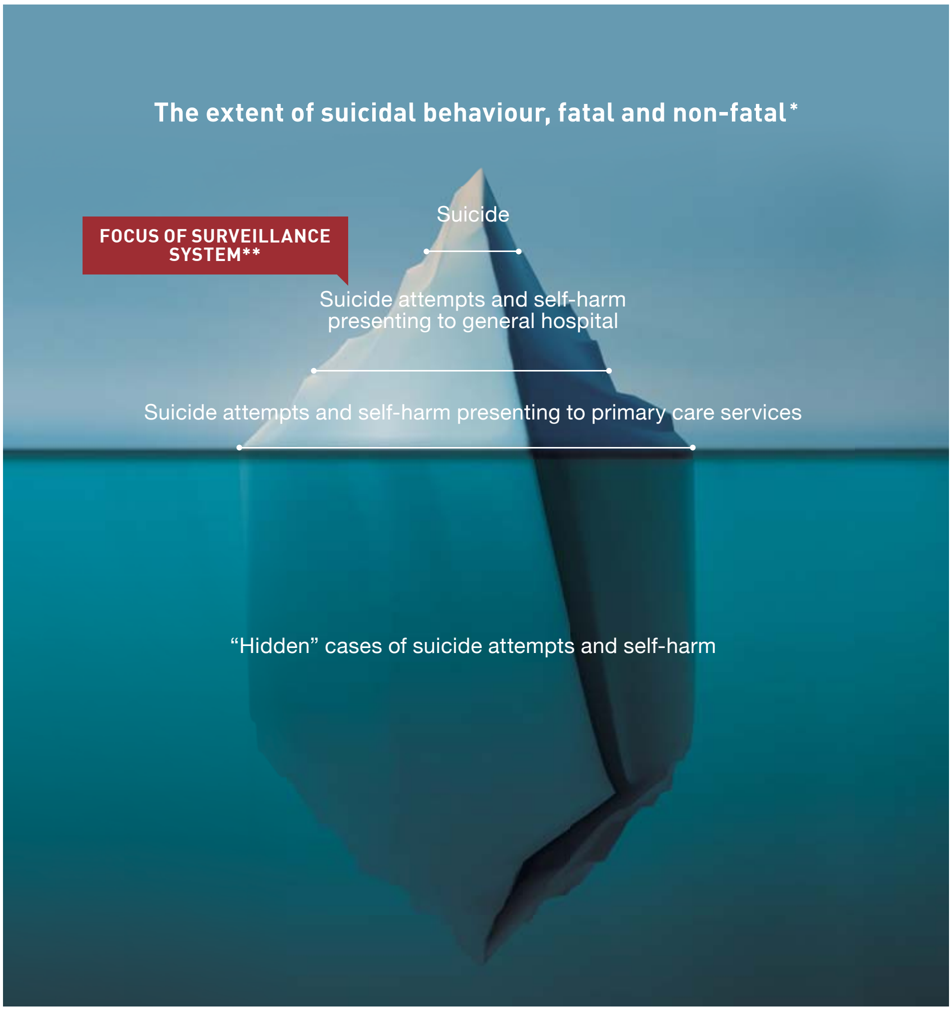

* Proportions illustrated in this diagram stem from international research findings; however, these proportions may vary between countries and regions.

** While increased accuracy and uniformity of information is needed at all levels, the focus of this manual is on suicide attempts and self-harm presenting to general hospitals.

1.3 Benefits of surveillance systems for hospital-presented suicide attempts and self-harm

Information provided by surveillance data of hospital-presented suicide attempts and self-harm contributes to key areas in addressing knowledge gaps and improving service provision for individuals presenting to hospitals following attempted suicide and self-harm (Figure 1.2). The establishment of sustainable and long-term surveillance systems for hospital-presented suicide attempts and self-harm enables the identification of patients who present with repeated acts over time and with risk factors associated with repetition (25, 26, 19). Access to surveillance data on hospital-presented suicide attempts and self-harm at national level will provide information on specific service capacity and treatment requirements (27).

Specific examples of benefits of surveillance systems for hospital presentations of suicide attempts and self-harm include the following:

- Access to real-time data can inform the allocation of specialized nurses in emergency departments, prioritizing specific hospitals or emergency care centres on the basis of the volume of suicide attempts and self-harm (18).
- Surveillance information on trends in suicide attempts and self-harm and on demographic and clinical risk groups is fundamental for developing, implementing and evaluating national suicide prevention programmes (9, 28).
- Surveillance data on methods used in suicide attempts inform the development of task forces to address the use of specific medications based on observed patterns of intentional drug overdose (18).
- Data on the extent of repeated suicide attempts and self-harm by hospital and by geographical region can inform the planning and implementation of treatment programmes. In Ireland, for instance, dialectical behaviour therapy for persons with a pattern of frequent repetition of suicide attempts and self-harm was implemented using a stepped approach based on the volume of repeated suicide attempts across hospitals (18).

In order to pursue the key objectives of a surveillance system for hospital-presented suicide attempts, long-term sustainability is crucial (29). For instance, identification of suicide-attempt patients with a risk of long-term repetition, and their associated characteristics, requires that data are obtained on consecutive cases of hospital-presented suicide attempts over several years (19). Since attempted suicide and self-harm are associated with high levels of morbidity, as well as mortality by suicide and other causes of premature death, sustaining the surveillance of suicide attempts and self-harm as a routine part of the health information system should be a key priority that is endorsed and supported financially by national governments and ministries of health.

Figure 1.2
Benefits of surveillance systems for hospital-presented suicide attempts and self-harm

Surveillance systems for hospital-presented suicide attempts and self-harm

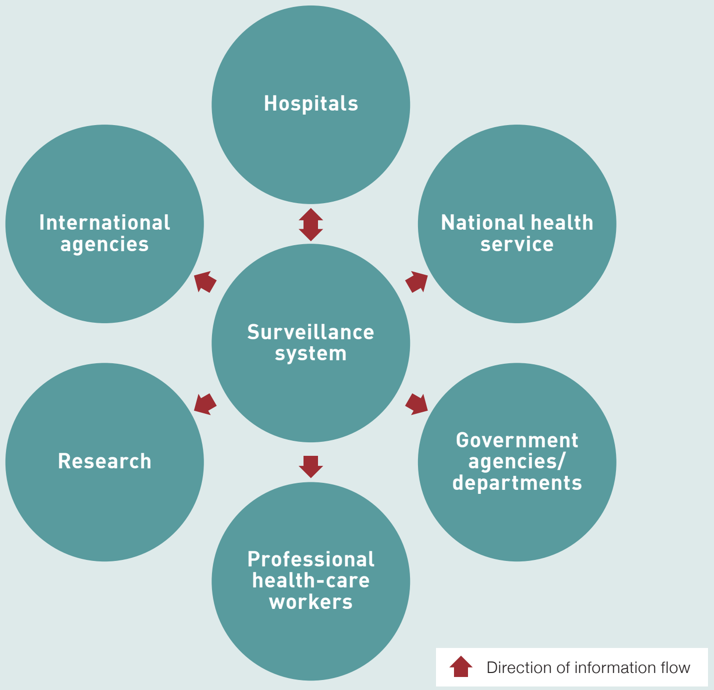

- Inform:
  - service provision, resource deployment and guidelines for management of suicide attempts and self-harm;
  - assessment of, and interventions for, suicide attempts and self-harm;
  - strategies and policies on clinical management of suicide attempts and self-harm.
- Provide: real-time data on all presentations to hospitals.
- Evaluate: guidelines, treatment and interventions.
- Measure: regional and demographic variations.

1.4 Aim of this manual

The aim of this manual is to improve standardization within and between countries with regard to establishing and maintaining a surveillance system of hospital-presented suicide attempts and self-harm. National and international access to accurate data on suicide attempts and self-harm will develop an understanding of factors influencing suicidal behaviour in each country, or area within a country, and will better inform public health policies on intervention and treatment as well as national suicide prevention strategies (18).

1.5 Who this manual is for

This manual is intended for health professionals, data registration officers, researchers and statisticians working at general hospitals or at university departments and research institutes, and those who are involved in accessing data relating to suicide attempts and self-harm from hospital records. The manual may also be of interest to other stakeholders, such as professionals working with ministries of health, decision-makers, and nongovernmental organizations.

1.6 Contents of the manual

The manual provides practical steps for the development and implementation of surveillance systems on suicide attempt and self-harm, based on medical records in hospitals. These practical steps also encompass reporting of surveillance outcomes and dissemination as well as maintenance and sustainability over time. The manual addresses training in a separate section where exclusion and inclusion criteria are discussed with vignette examples, and where vignettes for practice are provided. Furthermore, terminology is discussed because surveillance systems need consensus on terms, definitions and classification if they are to provide coherent surveillance information to the international dialogue. Consistency in terminology and definitions will facilitate comparison of the outcomes of surveillance systems on suicide attempts and self-harm at global level with regard to trends, risk groups and patterns of aftercare. Finally, a review of existing surveillance systems or surveillance projects for suicide attempts and self-harm is included since the surveillance of suicide attempts and self-harm has been increasingly prioritized at national and international levels in recent years. Data are drawn from dedicated registries, multicentre projects, national/international statistics and databases, and individual studies.

1.7 References

1. Hall IH, Correa A, Yoon P, Braden C. Lexicon, definitions and conceptual framework for public health surveillance. MMWR. 2012:10–4.
2. Preventing suicide: a global imperative. Geneva: World Health Organization; 2014.
3. Mental Health Action Plan 2013–2020. Geneva: World Health Organization; 2013.
4. Holder Y, Peden M, Krug E, Lund J, Gururaj G, Kobusingye O. Injury surveillance guidelines. Geneva: World Health Organization; 2001.
5. Sethi D, McGee K, Peden M, Habibula S. Guidelines for conducting community surveys on injuries and violence. Inj Control Saf Promot. 2004;11:303–6.
6. Bartolomeos K, Kipsaina C, Grills N, Ozanne-Smith J, Peden M, editors. Fatal injury surveillance in mortuaries and hospitals: a manual for practitioners. Geneva: World Health Organization; 2012.
7. Värnik P. Suicide in the world. Int J Environ Res Public Health. 2012;9:760–71.
8. Web-based Injury Statistics Query and Reporting System. Atlanta (GA): Centers for Disease Control and Prevention: 2014 (www.cdc.gov/injury/wisqars/index.html, accessed 18 January 2016).
9. Crosby A, Ortega L, Melanson C. Self-directed violence surveillance: uniform definitions and recommended data elements, Version 1.0. Atlanta (GA): Centers for Disease Control and Prevention, National Center for Injury Prevention and Control; 2011.
10. Bergen H, Hawton K, Waters K, Ness J, Cooper J, Steeg S, et al. How do methods of non-fatal self-harm relate to eventual suicide? J Affect Disord. 2012;136:526–33.

11. Carroll R, Metcalfe C, Gunnell D. Hospital management of self-harm patients and risk of repetition: systematic review and meta-analysis. J Affect Disord. 2014;168:476–83.

12. Gunnell D, Bennewith O, Peters TJ, House A, Hawton K. The epidemiology and management of self-harm amongst adults in England. J Public Health Oxf Engl. 2005;27:67–73.

13. Hawton K, Zahl D, Weatherall R. Suicide following deliberate self-harm: long-term follow-up of patients who presented to a general hospital. Br J Psychiatry J Ment Sci. 2003;182:537–42.

14. Owens D, Horrocks J, House A. Fatal and non-fatal repetition of self-harm. Systematic review. Br J Psychiatry J Ment Sci. 2002;181:193–9.

15. Arensman E, Kerkhof AJ, Hengeveld MW, Mulder JD. Medically treated suicide attempts: a four year monitoring study of the epidemiology in the Netherlands. J Epidemiol Community Health. 1995;49:285–9.

16. McMahon E, Keeley H, Cannon M, Arensman E, Perry IJ, Clarke M, et al. The iceberg of suicide and self-harm in Irish adolescents: a population-based study. Soc Psychiatry Psychiatr Epidemiol. 2014;49:1929–35.

17. Ystgaard M, Arensman E, Hawton K, Madge N, van Heeringen K, Hewitt A, et al. Deliberate self-harm in adolescents: comparison between those who receive help following self-harm and those who do not. J Adolesc. 2009;32:875–91.

18. Griffin E, Arensman E, Corcoran P, Dillon C, Williamson E, Perry IJ. National Self-Harm Registry Ireland. Annual Report 2014. Cork: National Registry of Deliberate Self-Harm; 2015.

19. Perry IJ, Corcoran P, Fitzgerald AP, Keeley HS, Reulbach U, Arensman E. The incidence and repetition of hospital-treated deliberate self-harm: findings from the world's first national registry. PloS One. 2007;7:e31663.

20. Bergen H, Hawton K, Waters K, Cooper J, Kapur N. Epidemiology and trends in non-fatal self-harm in three centres in England: 2000–2007. Br J Psychiatry. 2007;197:493–8.

21. Rajendra R, Krishna M, Majgi S, Heggere N, Robinson C, Poole R. A feasibility study to establish a Deliberate Self-harm Register in a state hospital in southern India. Br J Med Pract. 2015;8:807.

22. Ward E, McCartney T, Arscott-Mills S, Gordon N, Grant A, McDonald AH, et al. The Jamaica Injury Surveillance System: a profile of the intentional and unintentional injuries in Jamaican hospitals. West Indian Med J. 2010;59:7–13.

23. CIHI data access. Ottawa: Canadian Institute for Health Information; 2015 (https://www.cihi.ca/en/data-and-standards/access-data, accessed 18 January 2016)

24. Victorian State Trauma System and Registry: 1 July 2013 to 30 June 2014 summary report. Melbourne: Department of Health and Human Services of the Government of Victoria; 2015.

25. Griffin E, Arensman E, Wall A, Corcoran P, Perry IJ. National Registry of Deliberate Self-Harm Annual Report 2012. Cork: National Registry of Deliberate Self-Harm; 2013.

26. Lilley R, Owens D, Horrocks J, House A, Noble R, Bergen H, et al. Hospital care and repetition following self-harm: multicentre comparison of self-poisoning and self-injury. Br J Psychiatry J Ment Sci. 2008;192:440–5.

27. Griffin E, Arensman E, Corcoran P, Wall A, Williamson E, Perry IJ. National Registry of Deliberate Self-Harm Annual Report 2013. Cork: National Registry of Deliberate Self-Harm; 2014.

28. Health Service Executive. National Office for Suicide Prevention. Connecting for life: Ireland's national strategy to reduce suicide 2015–2020. Dublin: Department of Health; 2015.

29. Hawton K, Bale L, Casey D, Shepherd A, Simkin S, Harriss L. Monitoring deliberate self-harm presentations to general hospitals. Crisis. 2006;27:157–63.

02

Development and implementation of a surveillance system on suicide attempts and self-harm

Section overview

Implementing a surveillance system:
Outline of the requirements and steps in planning and preparing a surveillance system of hospital-presented suicide attempts and self-harm.

Data collection and case ascertainment:
Overview of how to inform stakeholders, the required data items and procedural aspects of data coding.

Ethics and data protection:
Accessing hospital data on suicide attempts and self-harm and associated patient characteristics, adherence to ethical guidelines and data protection procedures, and ensuring confidentiality.

Data management procedure:
- data collection, including registration forms and data entry;
- data compilation;
- data cleaning;
- data analysis;
- data interpretation.

Long-term surveillance:
The benefits of long-term surveillance in terms of access to annual data and its use in addressing relevant epidemiological, clinical and public health issues, including guidance on performing and interpreting statistical analyses.

Figure 2.1 provides an overview of five steps that are covered in the manual on setting up, implementing and then reviewing and evaluating a surveillance system.

Figure 2.1.
Stepped approach to developing and implementing a surveillance system for suicide attempts and self-harm

STEP 1 Inform and engage

- Inform and engage government/local government/relevant stakeholders
- Where possible, form an advisory committee
- Schedule time for regular meetings of all parties involved

STEP 2 Setting up the surveillance system

- Prepare a realistic budget
- Consider primary and secondary funding options, keeping in mind sustainability of the surveillance system
- Consider stepped implementation, including pilot phase and scaling up to the national level
- Appoint key function staff
- Develop standard operating procedures

STEP 3 Ethics and data protection

- Obtain ethical approval and formal written permission for data collection from hospital management
- Verify requirements from national data protection legislation

STEP 4 Implementation

- Enhance and refine standard operating procedures
- Conduct training in data collection (see Section 3)
- Develop data registration form
- Meet and fully brief hospital management and relevant staff from whom data will be obtained (e.g. emergency room staff and medical records staff)
- Coding and data entry
- Data compilation and data cleaning
- Data analysis and data interpretation
- Ensure all key staff are adequately trained

STEP 5 Review and evaluate

- Annual reporting and other formal reviews
- Review all aspects of the implementation of the surveillance system with all relevant parties
- Based on review, make necessary changes to management or standard operating procedures of the surveillance system
- Where appropriate, expand to additional regions/hospitals
- Ensure the maintenance and sustainability of the surveillance system

2.1 Informing and engaging governments and relevant stakeholders

STEP 1

Whether the scope of the surveillance system is national or subnational, it is suggested to involve representatives from the Ministry of Health and other relevant ministries during preparation and implementation. Although it is desirable to pursue the implementation of a national surveillance system from the outset, lessons from case examples of existing national or subnational registries of hospital-presented suicide attempts and self-harm (such as those in Ireland, Northern Ireland and Flanders [Belgium]), show that full coverage was achieved by scaling up implementation from one geographical area to further areas. In developing a surveillance system for suicide attempts and self-harm, countries can take a stepped approach, as outlined in Figure 2.1.

In line with the WHO report *Preventing suicide: a global imperative*, national surveillance of suicide and suicide attempts is considered a core element of national suicide prevention strategies. Therefore, in countries where a national suicide prevention strategy is currently being initiated, this may be a timely opportunity to propose simultaneous development and implementation of a national surveillance system for hospital-presented suicide attempts and self-harm (as in Bhutan, Mongolia or Tunisia where such a system is being both developed and implemented).

If possible and if the resources are available, countries can consider setting up the following groups:

1. A steering committee consisting of relevant government officials and others for the overall guidance and support of the surveillance system and the resources required.
2. A technical advisory group to provide guidance and technical inputs to the planning, implementation and evaluation of the system.
3. A surveillance management team (consisting of a data collector and system manager according to available resources) to develop, implement and review the surveillance system.

The steering committee, which should provide guidance to the technical advisory group and surveillance management team, should comprise representatives of the Ministry of Health, medicine, emergency medicine, public health, psychiatry, nursing, suicide prevention, psychology, health information systems and biostatistics. In the initial phase of developing and implementing the surveillance system, it may be helpful to invite an external advisor to the steering committee – particularly a professional with extensive expertise in developing and implementing a surveillance system for hospital-presented suicide attempts and self-harm. The technical advisory group can be a relatively small group representing professionals from relevant disciplines at local level and geared towards implementation of the surveillance system in a specific geographical region. Timelines for meetings should be determined according to factors specific to each surveillance system (such as geographical coverage and population).

In many countries, it will not be feasible to have three distinct groups as described. In this case, the best fit for the context and the resources should be considered when drawing a group of experts or others.

2.2 Setting up the surveillance system

STEP 2

2.2.1 Costs and potential funding sources

Establishing and maintaining a surveillance system for hospital-presented suicide attempts and self-harm requires substantial resources. Preparing a realistic budget and allocating funding is a key objective of planning. The budget will vary according to the scope of the surveillance system – i.e. national or subnational (regional) – in each country.

Essential items to include in a budget for a surveillance system for hospital-presented suicide attempts and self-harm are shown in Table 2.1.

Table 2.1. Template of a budget for the surveillance system

Item	
1. Personnel	• Full-time or part-time manager<br>• Full-time or part-time data collector(s),<br>• Full-time or part-time research staff/statistician
2. Travel	• For data collection purposes<br>• To attend training workshops
3. Training	• Training workshops
4. Monitoring and evaluation	• Quality assurance checks
5. Overheads	• Office space and associated costs<br>• Equipment, computers, servers, software etc.<br>• Information technology support
6. Dissemination	• Design and printing of annual report, bulletins, newsletters etc.<br>• Publishing costs

The costs of developing, implementing and maintaining a surveillance system for hospital-presented suicide attempts and self-harm will vary between countries due to variation in operational costs such as salaries and office facilities. In completing the budget it is recommended, where possible, to obtain information on surveillance systems and studies that are already underway elsewhere in order to determine whether the budget is realistic.

In line with existing surveillance systems of hospital-presented suicide attempts and self-harm, it is suggested to explore options for government funding as the primary funding source for the surveillance system. In countries with a national suicide prevention strategy (either implemented or under development) which typically receives funding from the Ministry of Health and co-funding from other government departments, it is likely that funds for the surveillance system will be provided. In the negotiation process with government representatives, it will be important to highlight the benefits of surveillance of hospital-presented suicide attempts and self-harm, and the benefits of treatment and prevention of attempted suicide, self-harm and suicide.

It is important to incorporate the budget into a detailed proposal for development and implementation, including a strategy for long-term maintenance of the system with clarification of the long-term benefits and cost-effectiveness. If core funding from the government is ensured, it is suggested to explore options for co-funding from other sources – such as national or regional health/public health research funding programmes – to support complementary functions, innovation and research related to the surveillance system. However, it is important to bear in mind that short-term and once-off funding will not be sufficient for establishing and maintaining a surveillance system.

2.2.2 Appoint key function staff

It is crucial to appoint a surveillance management team (national or regional, depending on what is possible in a country). The team will vary in size depending on the size of the country or area. At least one senior professional should be appointed to complete the following duties as the system manager:

- overall management of the surveillance team;
- establishment of links with the general hospitals and ministries involved;
- daily management of the surveillance system, including providing training and supervision to the staff members involved in data collection in hospitals;
- management and analysis of data.

The team should be able to consult with a biostatistician/statistician on statistical issues, including conducting appropriate statistical analysis, as required.

A data collector should be appointed. If resources are available, this role would ideally not be carried out by a member of the hospital staff. If resources are not available, data collection by hospital staff who have appropriate training and experience in working with health information systems may be appropriate in the initial stages of implementation. Internationally, there are examples of surveillance systems and studies of hospital-presented suicide attempts and self-harm in which hospital staff are involved in the data collection (1, 2, 3, 4). However, evaluations have shown that it is difficult to combine clinical duties with other tasks such as systematic data collection (3). In order to ensure a high level of standardization of data collection, the data collectors, whether hospital staff or external persons, should receive training in collecting data from medical records. There is further detail in Section 3 on training.

2.2.3 Standard operating procedures

Standard operating procedures should be developed in each country in line with the local or regional context to provide guidance on data collection, case ascertainment, data items and analysis processes for the surveillance system. This provides consistency for the surveillance system in a regional context.

2.2.3.1 Data collection

Data collectors are responsible for the collection of information on consecutive cases of self-harm, including suicide attempts, that present to hospital emergency departments. Where possible, each data collector will work within a number of hospitals (public and private) to gather and collate this information. The data collector will be responsible for the identification of cases from hospital records of incidences of self-harm and suicide attempts, the extraction of relevant information from these records, and the systematic recording of these anonymous data.

The principle duties of the data collector (or equivalent staff) include the following:

- to identify in a systematic way suicide attempts and self-harm from available hospital records (even when information is brief or basic) within a particular catchment area, using predefined inclusion and exclusion criteria (Section 3);
- to transfer these data securely to the system manager of the surveillance system at agreed intervals (e.g. once a month);
- to check, when appropriate, any coding queries and to perform quality control activities as and when required.

The data collector should work within the national guidelines of data protection and should sign a confidentiality agreement on appointment by the surveillance management team.

If a data collector is external, the following steps should be taken before a first visit to a hospital:

- Hospital management should meet representatives of the surveillance management team and should be fully informed about the surveillance system.
- Formal written permission for data collection should be obtained from the hospital's chief executive officer or manager.
- Relevant emergency department staff and medical records officers should be informed of the surveillance system.

A data collector's first visit to a hospital should be by appointment. Ideally, the hospital chief executive officer will personally introduce the data collector to the relevant hospital staff (e.g. emergency department, medical records personnel).

Visits to hospitals must be made regularly – weekly, fortnightly or monthly according to the number of attendances at the emergency department involving suicide attempts. Where practical, it is preferable to make a courtesy telephone call prior to visiting a hospital for data collection. It is useful to have contact with a member of staff who is regularly on duty (e.g. an administrative officer). In larger hospitals where weekly visits are needed, it may be possible to arrange a regular time slot that is convenient to hospital staff.

Procedures for the data collectors:

- Data collectors must make themselves known to hospital staff at each visit. Identification as surveillance system data collectors and/or authorization issued by the hospital must be carried at all times.
- Arrangements for security and confidentiality within the hospital must be strictly observed. Medical records should never be taken from areas assigned to data collectors without the specific permission/knowledge of hospital staff.
- Data collectors should always be aware of the current situation in an emergency department and should be prepared to leave if necessary.
- All written material pertaining to the surveillance system must be kept in the possession of the data collector.
- Personal details of cases should not be discussed.
- Any requests for information must be referred to the office of the national or regional surveillance system.
- Any material that is not relevant to the surveillance system should be ignored.
- Details of identifiable patients should never be discussed over the telephone.
- Data collectors should conduct themselves in a professional manner and use appropriate language.

2.2.3.2 Case ascertainment

In order to ensure a consistent and uniform approach to case ascertainment, similar procedures should be adhered to by all data collectors.

The data collectors must check all entries in the medical records, casualty book or the relevant hospital computer file that logs every presentation to the hospital. Most hospital presentations will occur through the emergency department, but systems should be put into place to check records of all presentations to the hospital. If the hospital does not permit the data collector to access the computer system, a printout must be sought that gives details of all presentations. If the hospital will provide such a printout only for specific types of emergency department presentations, it is important that the categories are broad enough to include as close to 100% of all intentional self-harm presentations as possible (according to the inclusion and exclusion criteria outlined in Section 3). Training guidance on how to recognize cases is included in Section 3.

If there is evidence (e.g. a note written in the medical records) that a patient did not want his/her data to be passed to an agency outside of the hospital, the patient's wish must be respected. In such a case, no information on this patient should be recorded by the data collector.

All cases of intentional self-harm that have presented to the hospital should be identified. The hospital number, date and time of attendance, along with other relevant details should be noted in the registration form or in the electronic data entry system. In principle, the data collector should spend as little time as possible at the casualty book or hospital computer. The relevant medical record or hospital chart should be consulted in order to confirm that the presentation meets the case-definition criteria of the surveillance system (Section 3).

In practice, if a data collector is unsure whether a hospital presentation is a case to be included or not, the collector should contact the system manager who should be able to provide further technical supervision and advice.

2.2.3.3 Data items

Ideally, core data items should be included in the registration form of the surveillance system on hospital-presented suicide attempts and self-harm. These core items should be consistent with those of major existing surveillance systems at the international level. In addition, a list of optional data items is proposed, which are relevant in terms of obtaining further detailed information on sociodemographic, psychosocial and psychiatric characteristics of the persons who have presented to hospital after a suicide attempt or self-harm.

Core data items in registration form

- Data collector (name or identification number of designated person collecting data)
- Date of registration
- Hospital number (a number should be assigned to each hospital when the surveillance system is being set up)
- Unique event number (each event of self-harm should be given a unique number)
- Unique person identification number (each person should be given a unique identification number)
- Sex
- Date of birth
- Age
- Postal/area code
- Date of presentation
- Time of presentation
- Mode of arrival at the hospital
- Seen by on arrival at the hospital
- Date of the self-harm
- Day of the week of the self-harm
- Time of the self-harm

- Location of the self-harm
- Method(s) according to ICD-10 codes (see Table 3.1). If the person has engaged in multiple methods of self-harm, all methods should be recorded; for intentional self-poisoning (X60–X69), the name and quantity of the substance should be provided
- Medical severity of the self-harm (medical severity would include one of the following: treatment in specialized unit, including Intensive Care Unit, Hyperbaric Unit, or Burns Unit; or surgery under anaesthesia; or extensive medical treatment, including antidotes for drug overdose or pesticide intoxication, telemetry or repeated tests or investigations; or objective evidence of altered level of consciousness, or unconscious, at or prior to presentation)
- Statement of intention to die (this information about suicidal intent is often not available or not reliable). If yes, provide a description of the statement
- History of self-harm (previous self-harm)
- Psychological/psychiatric assessment in the hospital
- Diagnosis (any diagnosis associated with the person)
- Admission to the hospital
- Discharge (day and time the person left the hospital)

Optional data items

- Nationality
- Country of origin
- Ethnicity
- Religion
- Marital status
- Employment status
- Socioeconomic status
- Sexual orientation
- Living circumstances / type of accommodation
- Referral
- Follow-up
- Previous admissions to hospital for self-harm
- Mental disorder / psychiatric history
- Alcohol or drug use
- Chronic pain and/or physical illness
- History of treatment for physical or mental health problems
- Acute emotional distress (e.g. break-up in relationship, family discord or loss, job loss, financial difficulties, housing problems, legal problems, problems at school or at work, intimate partner violence, experience of crime, trauma or abuse, being bullied, experiencing discrimination)
- Suicide or suicide attempt in the family
- Suicide or suicide attempt among friends, school-mates or work colleagues
- Knowledge of suicide or suicide attempt from the Internet or other media

2.3 Ethical requirements, confidentiality and data protection

STEP 3

Confidential data are any personal information relating to an individual that would make it possible to identify the person either directly or indirectly by reference to an identification number or by reference to one or more factors specific to her/his physical, physiological, mental, economic, cultural or social identity.

Data presented in statistical form, which are of such a type that the persons concerned can no longer be reasonably identified, are no longer considered to be confidential data, as all efforts have been made to ensure anonymity.

Confidentiality in the use of personal data in medical research is governed both by the ethical guidelines of the medical profession and by data protection legislation in each country. The principles of confidentiality must apply not only within the surveillance system but also to any data released by it, whether as public information or to individual researchers. As a safeguard, all surveillance system staff should be required to sign a confidentiality agreement at the time of their appointment, in addition to the confidentiality forms they have signed if they are hospital staff.

Preservation of confidentiality is not only an obligation but is also essential for maintaining the trust of those who provide the surveillance system with information. Hospital staff must be assured that the welfare of their patients will be respected and that the staff of the surveillance system will observe the same strict rules on confidentiality that exist in the hospital. The surveillance system staff also have legal and moral obligations to avoid acts that may cause suffering or distress to any individual.

In carrying out their functions, the surveillance system staff must adhere to the following:

- Permission to access medical records must be sought from the ethics committee of each individual hospital.
- An information leaflet describing the surveillance system should be made available in a suitable place within each participating hospital (see Annex 1 for an example).
- The surveillance system must cause no disadvantage, harm or distress to any person.
- Appropriate safeguards must be in place to preserve the confidentiality of the information in the custody of the surveillance system.
- Reports of the surveillance system must not contain information which could disclose any person's identity.

2.4 Implementation

STEP 4

In addition to the following items relevant to implementation, it is critical that staff are provided with the necessary training to ensure they understand their role, the data they are required to collect, and the relevance of their role within the overall process (see Section 3). Consequently, staff must be kept up to date on any new processes or developments and should be shown the value of their work.

2.4.1 Enhance and refine standard operating procedures

To ensure that all data to be included in the surveillance system are collected in a timely and accurate manner, it is necessary to do the following:

- Create standard operating procedures (SOPs) relevant to the context and update them annually.
- Ensure that all data collectors have an induction training programme.
- Hold regular meetings to ensure that the data collection is conducted in accordance with the operational criteria for ascertaining cases of self-harm and suicide attempts, and that the inclusion and exclusion criteria are respected.
- Host workshops for the data collectors (if there are several people) to help them share their learning and experiences, and to discuss how to handle challenging cases and situations, particularly in emergency departments.
- Ensure that reports, publications and updates on service developments (such as allocation of specialized self-harm assessment nurses in the emergency department) arising from the surveillance system are disseminated to the data collectors.

2.4.2 Develop a data registration form

Information on every case of self-harm should be either recorded on a paper data registration form or entered directly into an electronic database. Registration forms and electronic data entry systems should include all core data items and as many of the optional data items as is feasible (core and optional data items are listed in section 2.2.3.3).

Where possible, data items should be organized in drop-down menus to increase efficiency. For example:

- For the data item specifying the method of intentional self-harm, the ICD-10 codes should be listed (X60–X84), with an "other" category allowing for specification of other methods.

When necessary, as in the case of method of self-harm, an option must be given for selection of multiple answers.

An example of a paper data registration form is found in Annex 2. An example of an electronic data entry system can be obtained from the National Self-Harm Registry Ireland[1].

[1] *See: http://nsrf.ie/our-research/our-systems/national-registry-of-deliberate-self-harm, accessed 18 January 2016.*

2.4.3 Coding and data entry

Where possible, data should be entered directly into an electronic database as this is the most efficient method of collating data. If this is not possible, paper data registration forms can be used to enter data initially so that the data can be subsequently entered into a computerized system. Data elements should be coded (e.g. 1=female, 2=male).

In order to monitor the repetition of suicide attempts and self-harm, a surveillance system should record data according to events/presentations/episodes as well as persons. A unique person identification number is necessary to ensure accurate calculation of the number of persons presenting to hospitals after a suicide attempt or self-harm. A unique event number is required to calculate the number of total presentations in a given time period (since several presentations may be made by the same person). In the case of a repeated presentation, a new event number should be generated but the same unique person identification number should be used.

2.4.3.1 Unique event number

An electronic data entry system can be programmed to generate a unique event number automatically as each new record is saved. For paper data registration forms a unique event number should be assigned to each copy of the registration form that is produced.

2.4.3.2 Unique person identification number

When data are entered into an electronic database or onto a paper data registration form, information that can directly or indirectly identify a person – such as a person's full name, health identification number or street address – must be coded in accordance with the data protection regulations of the country concerned. This is necessary to prevent a person's identity from being linked to the information collected.

As an example, the following coding could be applied to create a unique person identification number:

- Use the following elements and define a standardized system to combine them into a unique code to be used as the unique person identification number:
  - First and last name: Use, for example, the initials or the first two letters of the first name and the last two letters of the last name or the partial name of the person
  - Age: Use, for example, the person's age or age range (e.g. any age between 30 and 39 years is coded as 30)
  - Date of birth: Use, for example, the person's year of birth
  - Address: Use, for example, the person's postal code or partial postal code (such as the first two numbers of the postal code).

The result of a code from this example of a standardized system could be SH367810. The situation in which a given unique code appears twice or more often must be avoided so it is preferable to have the coding conducted automatically by an electronic data entry system which provides an alert when a unique code is created a second time. Alternatively, when using paper data registration forms, a pre-generated list of random codes could be used for unique person identification numbers. The list linking the unique person identification number to a person's medical record should be kept in a safe or log controlled by the hospital. This safe or log should be kept secure at all times; if electronic, it should be double password-protected and encrypted.

This standardized system of creating codes should be confidential and known only to those controlling the electronic database. Similarly, the list linking a person's identity to randomly generated codes through a paper system should also be protected.

Confidentiality in the use of personal data in medical records is governed by both the ethical guidelines of the medical profession and the data protection legislation in each country. Consequently, the method of coding must be informed by the data protection legislation of each country. Where data protection legislation does not allow for the recording of personal data without consent, an alternative anonymized coding procedure of hospital presentations of self-harm could be agreed with the designated local or national office for data protection.

2.4.4 Data compilation

The compilation of the data provides the first stage in the quality assurance process. Data compilation should be performed by the manager of the surveillance system in collaboration with the data collectors. This is an important stage in the process, as the system manager can monitor the progress of data collection and assess how up-to-date the data is.

To ensure that all surveillance system data is compiled in a systematic way:

- The system manager should oversee and support the data collectors so they are clear as to their function and responsibility.
- The system manager should ensure the timely receipt and compilation of data from the different sites.
- The system manager should ensure that regular data audits/cross-checking exercises are carried out (e.g. bi-annually) with the involvement of all data collectors.
- In some countries, where resources are finite, a single person may perform the tasks attributed to both the data collector and the data manager, although this is not ideal.

2.4.5 Data cleaning

The data cleaning should be performed by the system manager in collaboration with the data collector. This stage can occur at the same time as data compilation. Any missing values or variables can be queried with the data collector at this time in order to ensure that all possible information can be included. This may require the data collector to revisit some case files to check missing information. As a result, data cleaning should not be considered to be complete until the end of the year when all data have been processed for the annual report.

The help of a statistician can be useful in this process, if required, to ensure that all necessary data are present to perform statistical analysis. The system manager and the statistician should work in close communication during this stage.

It is important that:

- All discussions and decisions at this stage should be recorded for future reference (e.g. all cases removed during this stage should be documented). While this may be time-consuming, it is critical for ensuring the overall reliability of the data.

- Key lessons from this process should be fed into the standard operating procedures and back to the data collectors as part of continued personal development.

- Sometimes it is required to re-check data at a later stage. It is crucial that original data files are retained for such purposes.

2.4.6 Data analysis

Data analysis and maintenance of the central database should take place in parallel. Data should be processed and analysed quarterly. Every time one quarter's data have been processed, the quarter file is merged with previous quarters until a full-year file is created.

The quarterly analysis of the data allows for summaries to be compiled and disseminated. It also allows for the data to be checked for quality and consistency. Maintaining the master data file and databases is critical for any retrospective and prospective analysis.

The suggestions at this stage are as follows:

- The system manager should ensure that the database and working master file are maintained and that two versions are securely stored.

- The database and master file should be backed up at the end of each quarter-year to an external hard drive. The external hard drive must be encrypted and stored in a locked safe when not in use.

2.4.7 Data interpretation

This section provides information on both basic and advanced statistical analyses which are commonly used in analysing surveillance data on suicide attempts and self-harm.

The final stage of the process is the interpretation and report writing. As the final product, reports produced from the surveillance system should be written with the following points in mind:

- Report on actual presentations, trends and relevant comparisons, and highlight key areas to be reviewed/considered.
- Stimulate suggestions for peer review and academic papers, and note opportunities to actively engage in the writing of papers which should be open to all relevant staff.
- Inform health and social care bodies and other relevant agencies (e.g. police, prisons, communities).
- Use reports for promotion of suicide awareness to as wide an audience as possible.

2.4.7.1 Basic statistical analyses

Calculation of rates per 100 000 population

The annual incidence rate per 100 000 population should be calculated for the total population, for the male and female populations separately, and for subgroups by age and sex, based on the number of persons who presented to hospital following a suicide attempt or self-harm in each calendar year.

It is suggested that crude and age-standardized self-harm rates (including suicide attempts) should be calculated by dividing the number of persons who engaged in self-harm (n) by the relevant population figure (p) and multiplying the result by 100 000 – i.e. (n/p) x 100 000. Rates should be calculated on the basis of the number of persons resident in the relevant area who engaged in self-harm irrespective of whether they were treated in that area or elsewhere.

It is advisable to adjust for the age composition of the population under study as this ensures that differences observed by sex or by area are due to differences in the incidence of self-harm rather than differences in the composition of the populations. For instance, European age-standardized rates (EASRs) are the incidence rates that would be observed if the population under study had the same age composition as a theoretical European population. EASRs can be calculated as follows: for each five-year age group, the number of persons who engaged in self-harm is divided by the population at risk and then multiplied by the number in the European standard population (5). The EASR is the sum of these age-specific figures. This can be done

for different regions and also globally using the same method.
The following points should be kept in mind:

- Calculated rates that are based on fewer than 20 events may be unreliable measures of the underlying rate.

- If the same individual presents to the hospital more than once on the same calendar day, it should be clarified whether a second suicide attempt or act of self-harm has been made or whether the re-presentation is due to absconding and returning, or being transferred to another hospital. If no second suicide attempt or act of self-harm has been made, this should be recorded as a single suicide attempt or self-harm event.

Confidence intervals

Confidence intervals provide a margin of error within which underlying rates may be presumed to fall on the basis of observed data. Confidence intervals assume that the event rate (n/p) is small and that the events are independent of one another. A 95% confidence interval for the number of events (n) is n +/- 2√n.

- For instance, if 25 acts of self-harm (including suicide attempts) are observed in a specific region in one year, the 95% confidence interval will be 25 +/- 2√25 or 15 to 35. Thus, the 95% confidence interval around a rate ranges from (n-2√n)/p to (n+2√n)/p, where p is the population at risk. If the rate is expressed per 100 000 population, these quantities must be multiplied by 100 000.

- A 95% confidence interval may be calculated to establish whether the two rates differ in statistical significance. The difference between the rates is calculated. The 95% confidence interval for this rate difference (rd) ranges from rd-2√(n1/p12+n2/p22) to rd+2√(n1/p12+n2/p22). If the rates were expressed per 100 000 population, then 2√(n1/p12+n2/p22) must be multiplied by 100 000 before being added to and subtracted from the rate difference. If zero is outside the range of the 95% confidence interval, the difference between the rates is statistically significant.

Repeat event analysis

Recording the unique person identification number allows for the analysis of repeated suicide attempts and self-harm acts. A repeated suicide attempt or act of self-harm should be defined as a re-presentation to any hospital due to a further suicide attempt or self-harm act undertaken after leaving the hospital following presentation for a previous suicide attempt or self-harm act. An example of repeat event analysis is conditional risk set analysis.

2.4.7.2 Advanced statistical analyses

Time series data and examining the impact of the economic recession on suicide attempts and self-harm

As an example of the values of time series data, an in-depth analysis including data from the National Self-Harm Registry Ireland found that, during the period 2008–2012, there was a negative impact of the economic recession on national rates of suicide and self-harm (including suicide attempts) in Ireland (6). In order to quantify the impact of this change, the study compared the observed rate of suicide and self-harm with the rate that would have been observed had the decreasing trend of suicide rates continued (i.e. if the rates of suicide and self-harm had continued to decline as they had been doing). Interrupted time series analysis (7) was performed to test if the recession had an impact on the level and trends of suicide and self-harm. The first time period of 2008 was taken as the advent of the recession. The analysis was repeated using two different recession starting points (Quarter 3/July 2007 and Quarter 3/July 2008) in order to assess the sensitivity of the results.

The results indicated that five years of economic recession and austerity in Ireland had a significant negative impact on rates of suicide in men and on self-harm in both sexes. The findings highlight the need to guide government responses to economic recessions in relation to suicidal behaviour and other health outcomes.

Time series data and examining the risk of repeated self-harm and suicide

Data collected in the Multicentre Study of Self-harm in England covered all self-harm hospital presentations by individuals aged 10–18 years between 2000 and 2007, and national death information on these individuals to the end of 2010 (8). Cox hazard proportional models were used to identify independent and multivariable predictors of repetition of self-harm (including suicide attempts) and of suicide.

Self-cutting as a method of self-harm in children and adolescents was reported to convey greater risk of suicide and repetition of self-harm than self-poisoning although different methods are usually used for suicide. The findings underline the need for psychosocial assessment in all self-harm cases presenting to general hospital.

Time series data and examining the trends in suicide attempts and changes in risk factors

Data from the USA's National Hospital Ambulatory Medical Care Survey included all visits for attempted suicide and self-inflicted injury (E950–E959) during 1993–2008 (9). Trend analyses were conducted using nptrend (a nonparametric test for trends that is an extension of the Wilcoxon rank-sum test) and regression analyses. A two-tailed $P<.05$ was considered statistically significant.

The findings showed that the volume of emergency department visits for attempted suicide and self-inflicted injury increased over the previous two decades in all major demographic groups. Awareness of these longitudinal trends may assist efforts to increase research on suicide prevention. In addition, this information can be used to inform current emergency department interventions and treatment programmes related to suicide and self-injury.

2.5 References

1. Caldera T, Herrera A, Renberg ES, Kullgren G. Parasuicide in a low-income country: results from three-year hospital surveillance in Nicaragua. Scand J Public Health. 2004;32:349–55.
2. Griffin E, Arensman E, Corcoran P, Dillon C, Williamson E, Perry IJ. National Self-Harm Registry Ireland, Annual Report 2014. Cork: National Registry of Deliberate Self-Harm; 2015.
3. Hawton K, Bale L, Casey D, Shepherd A, Simkin S, Harriss L. Monitoring deliberate self-harm presentations to general hospitals. Crisis. 2006;27:157–63.
4. Rajendra R, Krishna M, Majgi S, Heggere N, Robinson C, Poole R. A feasibility study to establish a Deliberate Self-harm Register in a state hospital in southern India. Br J Med Pract. 2015;8:807.
5. Revision of the European Standard Population: Report of Eurostat's task force. Luxembourg: European Union; 2013 (http://ec.europa.eu/eurostat/documents/3859598/5926869/KS-RA-13-028-EN.PDF/e713fa79-1add-44e8-b23d-5e8fa09b3f8f, accessed 18 January 2016)
6. Corcoran P, Griffin E, Arensman E, Fitzgerald AP, Perry IJ. Impact of the economic recession and subsequent austerity on suicide and self-harm in Ireland: an interrupted time series analysis. Int J Epidemiol. 2015;44:969–77.
7. Wagner AK, Soumerai SB, Zhang F, Ross-Degnan D. Segmented regression analysis of interrupted time series studies in medication use research. J Clin Pharm Ther. 2002;27:299–309.
8. Hawton K, Bergen H, Kapur N, Cooper J, Steeg S, Ness J, Waters K. Repetition of self-harm and suicide following self-harm in children and adolescents: findings from the Multicentre Study of Self-harm in England. J Child Psychol Psychiatry. 2012;53:1212–9.
9. Ting SA, Sullivan AF, Boudreaux ED, Miller I, Camargo CA. Trends in US emergency department visits for attempted suicide and self-inflicted injury, 1993–2008. Gen Hosp Psychiatry. 2012;34:557–65.

03

Training of staff involved in data collection

Section overview

Definitions:
Discussion on the importance of using consistent definitions for surveillance systems, and the challenges to setting uniform definitions.

Inclusion and exclusion criteria:
Helping data collection staff to identify cases and non-cases of suicide attempts and self-harm.

Practical vignettes:
Application of inclusion and exclusion criteria using case examples and solutions, and description of the rationale for ascertaining whether a situation is a case or not.

Context specific vignettes:
Recognition of cultural factors that can influence the method chosen.

3.1 Definitions

Over the years, a wide range of different terms have been used to indicate varying types of intentional self-harm. A prominent definition, which was initially developed for use in the WHO European regional multicentre study on non-fatal suicidal behaviour during the 1990s and has since been used in several surveillance systems and studies across different countries, is the following:

"An act with non-fatal outcome in which an individual deliberately initiates a non-habitual behaviour that, without intervention from others, will cause self-harm, or deliberately ingests a substance in excess of the prescribed dosage, and which is aimed at realizing changes that the person desires via the actual or expected physical consequences" (1).

De Leo et al. (2) simplified this definition as follows:

"A non-habitual act with non-fatal outcome that the individual, expecting, or taking the risk, to die or to inflict bodily harm, initiated and carried out with the purpose of bringing about wanted changes" (2).

For the purpose of the WHO report *Preventing suicide: a global imperative*, the term "suicide attempt" was used to mean *"any non-fatal suicidal behaviour"* and referred to *"intentional self-inflicted poisoning, injury or self-harm which may or may not have a fatal intent or outcome" (3).*

Review and classification of cases of suicide attempts and intentional self-harm are facilitated by using operational criteria. As the Platt et al. (1) definition provides relevant

details of the behaviour, intent and motivation, it is used for the purpose of standardizing operational criteria.

In accordance with this definition, the inclusion criteria refer to cases of intentional self-harm with varying levels of suicidal intent and varying underlying motives and cases of suicide attempts characterized by high levels of suicidal intent.

3.2 Exclusion and inclusion criteria

On the basis of the definition, the primary focus needs to be a description of what the individual is doing, without attempting at an initial or non-diagnostic stage to try to attribute or link motive or intent to what has occurred.

At the most basic level, for an individual presenting at the hospital, the questions to ask are the following:

- Was this injury or poisoning self-inflicted?
- If yes, was it intentional or accidental?

If the injury or poisoning is identified as intentionally self-inflicted on the basis of the exclusion and inclusion criteria (see Figure 3.1), the individual involved will require a psychological and/or psychiatric assessment. It is only at this stage that motivation and intent associated with the self-inflicted injury or poisoning can be explored.

Figure 3.1. Exclusion and inclusion decision-making

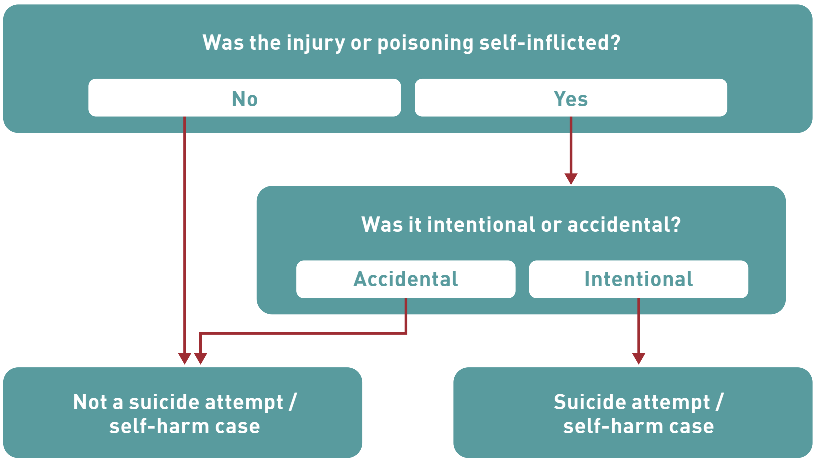

3.2.1 Exclusion criteria

On the basis of the definition, the exclusion criteria are as follows (i.e. the following are not considered to be cases of intentional self-harm or suicide attempt):

- **Accidental overdose of alcohol:** An individual who drinks alcohol to excess requiring hospital treatment, but without any intention to self-harm, and who does not combine alcohol with other methods of self-harm.

- **Accidental overdose of illicit drugs:** An individual who takes illicit drugs (e.g. cocaine, heroin, ecstasy) on a regular basis without any intention to self-harm.

- **Accidental overdose of prescription or over-the-counter medications:** An individual who incorrectly follows a prescribed dosage, or who takes additional medication in the case of illness, or who takes an excess of over-the-counter medication without any intention to self-harm.

- **Individuals who are dead on arrival at the hospital.**

3.2.2 Inclusion criteria

On the basis of the definition, the inclusion criteria are as follows (i.e. the following are considered to be cases of intentional self-harm or suicide attempt):

- All methods of intentional self-harm (as per ICD-10 coding, Table 3.1) (e.g. alcohol overdose, illicit drug overdose, ingestion of pesticides, laceration, attempted drowning, attempted hanging, gunshot wound) where it is clear that the self-harm was intentionally inflicted.

- All individuals who are alive presenting or referred to the hospital following an act of intentional self-harm or suicide attempt.

Some individuals may use a combination of methods, such as overdose of medication together with self-cutting. If the individual has engaged in multiple methods of intentional self-harm at the time of presentation, all methods should be recorded.

Examples of exclusion (not cases of self-harm or attempted suicide)

- Individual put his/her foot through the door in anger.
- Heavy intake of alcohol and medication (e.g. person withdrawing from heroin took a mixture of alcohol and medication hoping for relief).
- Person took usual medication twice by accident or to relieve chronic back pain.
- Insomnia-related incidents where medications are taken to help induce sleep.
- Medication taken to induce abortion for the purpose of terminating pregnancy.
- Alcohol-induced coma as a result of excessive alcohol intake by a young person experimenting with alcohol.
- Alcohol-induced coma as a result of excessive alcohol intake by a person suffering from alcohol dependence.
- Person had thoughts of drowning by jumping off a bridge, but took no action.
- Person is feeling unable to cope with suicidal thoughts.

Table 3.1.
ICD-10 codes for intentional self-harm (X60–X84)

X60 Intentional self-poisoning by and exposure to nonopioid analgesics, antipyretics and antirheumatics
X61 Intentional self-poisoning by and exposure to antiepileptic, sedative-hypnotic, antiparkinsonism and psychotropic drugs, not elsewhere classified
X62 Intentional self-poisoning by and exposure to narcotics and psychodysleptics [hallucinogens], not elsewhere classified
X63 Intentional self-poisoning by and exposure to other drugs acting on the autonomic nervous system
X64 Intentional self-poisoning by and exposure to other and unspecified drugs, medicaments and biological substances
X65 Intentional self-poisoning by and exposure to alcohol
X66 Intentional self-poisoning by and exposure to organic solvents and halogenated hydrocarbons and their vapours
X67 Intentional self-poisoning by and exposure to other gases and vapours
X68 Intentional self-poisoning by and exposure to pesticides
X69 Intentional self-poisoning by and exposure to other and unspecified chemicals and noxious substances
X70 Intentional self-harm by hanging, strangulation and suffocation
X71 Intentional self-harm by drowning and submersion
X72 Intentional self-harm by handgun discharge
X73 Intentional self-harm by rifle, shotgun and larger firearm discharge
X74 Intentional self-harm by other and unspecified firearm and gun discharge
X75 Intentional self-harm by explosive material
X76 Intentional self-harm by smoke, fire and flames
X77 Intentional self-harm by steam, hot vapours and hot objects
X78 Intentional self-harm by sharp object
X79 Intentional self-harm by blunt object
X80 Intentional self-harm by jumping from a high place
X81 Intentional self-harm by jumping or lying in front of moving object
X82 Intentional self-harm by crashing of motor vehicle
X83 Intentional self-harm by other specified means
X84 Intentional self-harm by unspecified means

3.3 Exclude or include?

The points below comprise exclusion and inclusion criteria and can be used to help make decisions as to whether cases should be included or excluded.

In all instances, these examples refer to information present in hospital records. Consequently, details may vary to reflect different levels of detail recorded in the medical records and system in place. Training to improve medical record-taking may be implemented, as necessary. Some examples of common abbreviations that may be found in medical records are as follows (see also examples below): BIBA – brought in by ambulance, C2H5OH – alcohol, GCS – Glasgow Coma Scale.

It is also acknowledged that surveillance systems depend on the information available. In some jurisdictions the system may be based on information from emergency room records only and no further information may be available to help decide on cases which are difficult to classify.

Where further information would be necessary to classify a case, the data collector should consult with the system manager. A policy should be in place regarding what steps to take in such situations.

Exclude, if the person's action or behaviour is the result of an accident

For example:

- accidental overdose (i.e. heavy episodic drinking or intake of illicit drugs, or as an unintended consequence of dependence on alcohol or illicit drugs);
- accidentally taking an incorrect dose of prescription medication;
- accidentally ingesting a poisonous substance;
- accidental injury related to sports or work;
- accidental injury as a result of an argumentative confrontation.

If it is clearly the result of an accident, then the case is not intentional self-harm or suicide attempt

ACTION **Exclude**

Vignette example – Accidental injury

Admission notes: 15-year-old boy BIBA. Injury to neck and laceration to leg. Mother accompanying. Says she found son hanging from window blind cord. Foot went through glass window. Explains she had asked him to clean windows. Heard a noise and breaking glass.
Behaviour: In shock. Says he slipped. Shivering and upset.

Exclude, if the patient's behaviour is related to a profound intellectual disability

For example:

– the person has no conscious awareness that he/she is self-harming.

This would apply *only* to patients with severe intellectual disability. Patients with milder intellectual disabilities can and do consciously self-harm (4).

If the disability is clearly severe and the person is not aware of the self-harming behaviour, then it is not a case of intentional self-harm or attempted suicide

ACTION **Exclude**

Vignette example – Intellectual disability

Admission notes: 28-year-old man with head injury. Profoundly autistic, accompanied by carer who explains he has a pattern of head-banging. **Behaviour:** Not communicative. No eye contact. Rocking back and forth and reluctant to allow head examination.

Include, if the person's behaviour is demonstrating suicidal intent

This would apply if any of the following are answered with "yes":

- Did the person leave a suicide note?
- Was the act carried out in isolation?
- Did the method used require planning and/or effort?
- Were precautions taken to avoid discovery?
- Without intervention would the chosen method have resulted in death?
- Is the person expressing a desire to die?
- Is the person refusing to cooperate with treatment?

Assessment of suicide intent can be assisted by – but does not solely depend on – the administration of scales, some of which are the following: Beck Scale for Suicide Ideation (5), Reasons for Living Inventory (6), Suicide Probability Scale (7), Columbia Suicide Severity Rating Scale (8). For further reading on intent, see references (9, 10, 11, 12).

**If there are indications that the action was intentional or planned in advance, or the person is making a statement of intentionality,
then it is a case of intentional self-harm and could also possibly be attempted suicide**

ACTION Include

Vignette example – Suicidal intent

Admission notes: 49-year-old female semi-conscious. Brought in by her husband. Found empty packets of Olanzapine 10mg x 20, Venlafaxine 3g, plus empty strip of paracetamol (500mg x 36). Husband also recovered suicide note and he reports that this is a second attempt.
Behaviour: Withdrawn but calm. Refusing treatment. Says she did not want to be found and best for everyone if she is allowed to die.

Include, if a motive for intentional self-harm or attempted suicide is indicated
NOTE: Is the patient communicating?

Unless the patient communicates it is very difficult to know what reasons are behind the actions taken.

Examples of motives:

- **Suicidal intent:** "Leave me alone. I just want it to be over."
- **Ambivalent:** "I'm exhausted. I thought this would be fast or easy, but I can't even get killing myself right."
- **Cry of pain (wanting relief/escape from an unbearable state of mind):** an internalized emotional response to feelings about self and life in relation to self (13)

– expressing feelings of desperation;
– feelings of self-hatred;
– desire to self-punish;
– to feel sense of control.

- **Cry for help (acting to change a life or relationship situation):** an externalized emotional response to feelings about life events that impact on self (13)

– to get attention/love;
– to punish;
– to frighten;
– to test another person's feelings;
– to express an emotion;
– to get revenge.

If one or more of the above motives are communicated, then it is a case of intentional self-harm and should be recorded in the surveillance system; the case may also possibly be a suicide attempt, but sufficient information to confirm the level of suicidal intent may not be available.

ACTION Include

Vignette examples – Indication of motive (a)

Admission notes: 35-year-old man brought in by social worker. Intoxicated (C2H5OH) and suspected overdose as found with 5 empty 100 ml bottles of Calpol (liquid paracetamol). HIV+.
Behaviour: Sweating and nauseous, expressing suicidal intent due to HIV status*

ACTION Include

Vignette examples – Indication of motive (b)

Admission notes: 50-year-old male farmer BIBA semi-conscious. Discovered by relative. Empty alcohol bottles and container of carbamate next to bed. Unclear how much pesticide has been consumed.
Behaviour: Says no point in anything, that he is tired of living*. Intoxicated. Telling staff to go away and leave him alone.

ACTION Include

Vignette examples – Indication of motive (c)

Admission notes: 18-year-old female brought in by husband following ingestion of paraquat and chloroquine. According to husband she had argument with mother-in-law. Poured paraquat into glass and drank some with handful of chloroquine tablets before he was able to stop her. Not sure how much she drank but thinks about a quarter of a glass.
Behaviour: Says that she is angry with her mother-in-law and wants to teach her a lesson*. When husband is present seems to be happy.

* Communicated motive is underlined

Include, if there is information from past history of self-harm or suicide attempts

- Is there a record of this person engaging in previous acts of intentional self-harm or attempted suicide?
- Does the person have old scars or injuries?

Research shows that previous history of self-harm or suicide attempts is a risk factor for suicide, especially in the first 12 months following the self-harm act or suicide attempt (14, 15).

If one or more of the above items are communicated, then it is a case of intentional self-harm

ACTION ▶ **Include**

Vignette example – Past history of self-harm

Admission notes: 38-year-old female presents with deep cut to upper thigh. Self-presented as couldn't stop the bleeding and felt she needed stitches.
Behaviour: Calm and apologetic. Says she knows what she has done. Thinks she got stressed and pressured at work and needed the release. Has had two prior admissions for self-cutting and prior diagnosis of mood disorder.

The patient acted impulsively

Impulsive: acting momentarily; doing things suddenly without careful thought or planning.

For example:

– reckless action taken following the consumption of alcohol and/or illicit drugs
– a sudden act following a fight and/or emotional upset;
– a sudden act during a period of mania or hyperarousal associated with mental illness.

When looking at impulsivity, consider the following:

- There is still debate concerning the relationship between trait impulsivity and impulsivity of the act itself (16, 17, 18). Conner (16) defines impulsive suicide attempts as *acts of self-harm involving little preparation or premeditation*; non-impulsive suicide attempts are *preceded by preparation and forethought.*
- While some research indicates that there may be an interaction between alcohol use (intoxication) and impulsivity in relation to suicide risk, a recent review by Rimkeviciene et al. (18) found little support for this hypothesis.

- The association between impulsivity, aggression and suicidality is well documented and yet is still poorly understood (17).

Therefore, the possibility a person has engaged in self-harm or a suicide attempt should be considered when looking at impulsive acts that cannot be readily classified as accidental.

ACTION **Impulsivity is difficult to classify. If in doubt, consult with system manager**

The following vignette demonstrates an impulsive act of intentional self-harm in reaction to a stressful event.

Vignette example - Impulsive act: a case of intentional self-harm

Admission notes: 20-year-old man BIBA following road traffic accident. Witness who called ambulance reported that the car suddenly swerved across the road and drove straight into a wall.
Behaviour: Unconscious on admission but was conscious at the scene. Ambulance crew report that he said he was arguing on the phone with his girlfriend and she ended the relationship. Said he just didn't want to feel anymore and aimed his car at the wall.

The following vignette demonstrates a very clear impulsive act driven by anger where the patient, in a moment of rage, impulsively lashes out in an act that results in self-harm but could not be regarded as intentional self-harm or a suicide attempt.

Vignette example - Impulsive act: not a case of intentional self-harm

Admission notes: 49-year-old man presents with injury to right hand. Suspected fracture – index and middle finger and possibly thumb. Says he punched wall following fight with girlfriend.
Behaviour: Laughing but apologetic. Says he has a bit of a temper.

The next vignette presents a scenario that is more difficult to classify.

Vignette example - Impulsive act: possibly a case of intentional self-harm or suicide attempt

Admission notes: 25-year-old man BIBA following rescue from river at 2am in morning. People exiting a nightclub saw a man in the river nearby. Called ambulance and threw a life preserver to the man which he caught. Rescue services noted that he was intoxicated and hypothermic.
Behaviour: Monosyllabic. Says he was going for a swim. When asked who he would like staff to contact, said he didn't know anyone locally and didn't want family contacted as they lived very far away.

This may be a case but more information than admission notes alone would be required to determine whether it is a case of intentional self-harm or not.

3.4 Vignettes for practice

A series of case vignettes drawn from real-life hospital emergency department scenarios, and incorporating a range of cultural backgrounds and practices are used here to illustrate cases of intentional self-harm and suicide attempt. Cases that are more difficult to assess and non-cases are also included.

Note: *Medical terms can vary in different jurisdictions. Therefore medical terms and abbreviations used for the purposes of these examples may not exactly match terms used in a given country.*

The vignettes included in this section can be classified as examples of:
– cases of intentional self-harm;
– cases of suicide attempt;
– non-cases;
– ambiguous cases where more information would be required.

Each vignette is presented with additional information and ICD-10 coding (Table 3.1) where applicable.

The “Explanation” section is intended to offer an explanation based on the information provided in the selected cases. Consequently it sometimes refers to practices specific to various cultural backgrounds as a reminder that all countries and territories will have their own cultural traditions and patterns that may need to be considered when collecting data.

A space is left for the user to add additional notes or observations.

Cases of both intentional self-harm and suicide attempt should be included in the surveillance system.

Examples of cases of intentional self-harm and suicide attempt

Vignette 1	
**Admission notes:** 17-year-old girl presents with abdominal pain, pain in left shoulder and left chest, breathing difficulty and nausea. Began to vomit and get faint at home. Her mother looked at stomach and saw extensive bruising. Reports that daughter admitted she had been punching herself in the stomach.<br><br>**Behaviour:** Frightened and crying. Seems very anxious. Keeps saying "I'm sorry".	
**Example of:**	Intentional self-harm
**Explanation**	Self-punching is a method of self-harm. While the patient is expressing remorse, the behaviour was discovered due to the impact of injury rather than disclosure by the patient or any request for help from patient.
**ICD-10**	X-79
**Action**	Include
**Additional notes (user area)**	

Vignette 2	
**Admission notes:** BIBA. Man 35/40 years approximately. Emergency services called by ex-girlfriend whom he had texted. Polypharmacy overdose, some foreign language medications and empty vodka bottles, bruising to neck, rope found on bed. GCS 15/15.<br><br>**Behaviour:** Incoherent. Speaking in own language and seems to have poor grasp of local language.	
**Example of:**	Suicide attempt
**Explanation**	Act involved preparation and planning. Act was communicated to ex-girlfriend, so an awareness and admission of motivation is present.
**ICD-10**	X-61, X-65, X-70
**Action**	Include
**Additional notes (user area)**	

Vignette 3	
**Admission notes:** Elderly woman (approximately 75 years) BIBA. Couple saw her walking into the sea and called emergency services. Rescue services recovered the lady. Had a bag containing large stones strapped on back.<br><br>**Behaviour:** No eye contact, refusing to speak or provide any family details.	
**Example of:**	Suicide attempt
**Explanation**	Act involved preparation and planning. Lack of communication means that motivation cannot be assessed.
**ICD-10**	X-71
**Action**	Include
**Additional notes (user area)**	

Vignette 4	
**Admission notes:** 19-year-old woman admitted with self-inflicted burns. Used paraffin to set fire to herself. Family member used fire blanket and called emergency services.<br><br>**Behaviour:** Semi-conscious, distressed and in pain.	
**Example of:**	Suicide attempt
**Explanation**	Self-immolation is a method frequently used in low- and middle-income countries in particular. Notes here state that she set fire to herself, implying that this information was provided to the emergency services by someone present at the time of the act.
**ICD-10**	X-76
**Action**	Include
**Additional notes (user area)**	

Vignette 5	
**Admission notes:** 19-year-old girl BIBA following ingestion of pesticide. Family member explains that she had been the victim of a sexual assault and that her boyfriend's family now does not want her.<br><br>**Behaviour:** Distressed, semi-conscious, in brief lucid period expressed wish to die.	
**Example of:**	Suicide attempt
**Explanation**	Ingesting pesticide is unlikely to be accidental, and suicidal intent is being expressed. Family are providing additional detail regarding possible motivation.
**ICD-10**	X-68
**Action**	Include
**Additional notes (user area)**	

Vignette 6	
**Admission notes:** 22-year-old male brought to the emergency room by roommate in a boarding house. Knowing that the patient was getting ready for school but complained of severe headache, informant went back to the boarding house after the patient failed to show up for the first class and did not answer phone calls. The patient was found lying on the bed, unconscious, with an empty silver cleaner bottle on the floor. Informant said the patient mentioned not being able to pay the last instalment of his tuition fee.<br><br>**Behaviour:** Unconscious	
**Example of:**	Intentional self-harm (unclear whether suicide attempt since information on suicidal intent is not known)
**Explanation**	Ingesting a chemical substance is unlikely to be accidental. Friend is providing additional detail regarding possible motivation.
**ICD-10**	X-69
**Action**	Include
**Additional notes (user area)**	

Examples of cases that are not intentional self-harm or suicide attempt

Vignette 7	
**Admission notes:** 21-year-old woman self-admitting. Took 6 solpadeine tablets for menstrual pain, denies self-harm intent, vitals stable.<br><br>**Behaviour:** Says she was in bad pain, took 2 tablets and then fell asleep and took more 30 minutes later.	
**Example of:**	Non-case
**Explanation**	Self-presenting combined with concern for own health and quantity taken.
**ICD-10**	Not applicable
**Action**	Exclude
**Additional notes (user area)**	

Vignette 8	
**Admission notes:** Recently widowed lady presents with minor burns to large body area. Denies self-harm. Says her in-laws made her do it due to an inheritance dispute but she ran and rolled on ground. Sister helped her get to hospital.<br><br>**Behaviour:** Nervous and quiet. Is afraid of in-laws.	
**Example of:**	Non-case
**Explanation**	While it is an alarming case which could imply a criminal act towards the patient, it is not an act of self–harm and her sister's account combined with cultural background verifies her story.
**ICD-10**	Not applicable
**Action**	Exclude
**Additional notes (user area)**	

Vignette 9	
**Admission notes:** 65-year-old woman BIBA following smoke inhalation. Discovered unconscious by family arriving for lunch. At scene GCS 12 now GCS 14. Home barbecue found alight in kitchen.<br><br>**Behaviour:** Says it began to rain so she thought grandchildren would enjoy indoor barbecue.	
**Example of:**	Non-case
**Explanation**	The use of charcoal barbecues is a suicide method, e.g. in China. However, in this instance the circumstances do not indicate that this was anything more than an accident.
**ICD-10**	Not applicable
**Action**	Exclude
**Additional notes (user area)**	

Examples of ambiguous cases that would require further information to ascertain whether they are intentional self-harm or suicide attempt

Vignette 10	
**Admission notes:** 22-year-old girl presents with severe abdominal skin infection. Recent cuts and old scarring on her abdomen. Denies self-harm.<br><br>**Behaviour:** Says that she is just following the custom of her tribe and it makes her beautiful.	
**Example of:**	Ambiguous case
**Explanation**	Ritualized scarring is a tribal custom in area. More information would be required as to whether the extent of scarring in this instance indicates suicidal behaviour.
**ICD-10**	If a case, then code as X-78
**Action**	Seek more information or consult with system manager for guidance.
**Additional notes (user area)**	

Vignette 11	
**Admission notes:** 19-year-old male BIBA with bullet wound to the leg. Accompanied by police who say he was pointing a gun at them and using threatening language. Refused to drop weapon so was shot in leg. Gun has been removed and was an airgun.<br><br>**Behaviour:** Agitated and still swearing. Restrained by handcuffs. History of admission for bipolar depression.	
**Example of:**	Ambiguous case
**Explanation**	This may be an intentional act and a case of attempted "suicide by cop" or it may be an unintentional act stemming from a history of mental illness.
**ICD-10**	If a case, then code as X-84
**Action**	Seek more information or consult with system manager for guidance.
**Additional notes (user area)**	

Vignette 12	
**Admission notes:** 32-year-old man BIBA and police. Fracture to both legs following fall from a building. Police have arrested him for attempted suicide. Say he is known to them and has made several suicide attempts in previous 2 months.<br><br>**Behaviour:** Denies self-harm. Says he fell and that police and authorities have been harassing him and spying on him.	
**Example of:**	Ambiguous case
**Explanation**	He is denying a suicide attempt and there are no witnesses. The police information regarding past history of suicidal behaviour may imply this was a suicide attempt. However, cultural issues have to be factored in. Firstly, he was arrested for attempted suicide, meaning that this is an area where suicide is still regarded as a criminal act and therefore the person may be likely to deny it. Secondly, if the cultural background is an oppressive one or one of civil unrest then the person's version of events may be true. More information is required.
**ICD-10**	If a case, then code as X-80
**Action**	Seek more information or consult with system manager for guidance.
**Additional notes (user area)**	

Vignette 13	
**Admission notes:** 48-year-old man BIBA. Bullet wound to the left cheek and cheekbone. Bleeding profusely. No other party involved.<br><br>**Behaviour:** Says he can't remember what happened. Must have been cleaning his gun.	
**Example of:**	Ambiguous case
**Explanation**	Without further information it would not be possible to categorize this as a case as the person is denying it and no witnesses or background information are available.
**ICD-10**	If a case, then code as X-72
**Action**	Seek more information or consult with system manager for guidance.
**Additional notes (user area)**	

Vignette 14	
**Admission notes:** 42-year-old female brought to the emergency room by a concerned citizen for bruises on face and arms. Woman was standing by the bridge, looking down towards the water underneath. She has no slippers on, has bleeding bruises on her face and arms.<br><br>**Behaviour:** At the emergency room, woman admits to have run away from home after her intoxicated husband beat her. Admits history of domestic violence in the past year. She refuses to provide any other information, particularly the contact information of the family.	
**Example of:**	Ambiguous case
**Explanation**	Without further information about the source of the bruises and why she is standing by the bridge it would not be possible to categorize this as a case.
**ICD-10**	Not applicable
**Action**	Seek more information or consult with system manager for guidance.
**Additional notes (user area)**	

3.5 References

1. Platt S, Bille-Brahe U, Kerkhof A, Schmidtke A, Bjerke T, Crepet P, et al. Parasuicide in Europe: the WHO/EURO multicentre study on parasuicide. I. Introduction and preliminary analysis for 1989. Acta Psychiatr Scand. 1992;85:97–104.
2. De Leo D, Burgis S, Bertolote J, Kerkhof A, Bille-Brahe U. Definitions of suicidal behaviour. In: De Leo D, Bille-Brahe U, Kerkhof A, Schmidtke A, editors. Suicidal behaviour: theories and research findings. Göttingen: Hogrefe & Huber Publishers; 2004:17–39.
3. Preventing suicide: a global imperative. Geneva: World Health Organization; 2014.
4. Buono S, Scannella F, Palmigiano MB, Elia M, Kerr M, Di Nuovo S. Self-injury in people with intellectual disability and epilepsy: a matched controlled study. Seizure. 2012;21:160–4.
5. Beck AT, Kovac M, Weissman A. Assessment of suicidal intention: the scale for suicide ideation. J Consult Clin Psych. 1979;47:343–52.
6. Linehan MM, Goodstein JL, Nielsen SL, Chiles JA. Reasons for staying alive when you are thinking of killing yourself: The Reasons for Living Inventory. J Consult Clin Psych. 1983;51:276–86.
7. Cull JG, Gill WS. Suicide Probability Scale. Los Angeles (CA): Western Psychological Services; 1992.
8. Posner K, Brown GK, Stanley B, Brent DA, Yershova KV, et al. The Columbia Suicide Severity Rating Scale (C-SSRS): initial validity and internal consistency findings from three multi-site studies with adolescents and adults. Am J Psychiatr. 2011;168:1266–77.
9. Arensman E, Keeley H. Defining intent. Psychiatry Professional. 2012;Spring:8–9 (http://ns-rf.ie/wp-content/uploads/journals/12/ArensmanKeeley(2012).pdf, accessed 18 January 2016).
10. Haw C, Casey D, Holmes J, Hawton K. Suicidal intent and method of self-harm: a large-scale study of self-harm patients presenting to a general hospital. Suicide Life-Threat. 2015;45(6):732–46.
11. Haw C, Hawton K, Houston K, Townsend E. Correlates of relative lethality and suicidal intent among deliberate self-harm patients. Suicide Life-Threat. 2003;33:353–64.
12. McAuliffe C, Arensman E, Keeley HS, Corcoran P, Fitzgerald AP. Motives and suicide intent underlying hospital treated deliberate self-harm and their association with repetition. Suicide Life-Threat. 2007;37:397–408.
13. Scoliers G, Portzky G, Madge N, Hewitt A, Hawton K, de Wilde EJ, et al. Reasons for adolescent deliberate self-harm: a cry of pain and/or a cry for help? Findings from the child and adolescent self-harm in Europe (CASE) study. Soc Psych Psychiatr Epid. 2009;44:601–7.
14. Hawton K, Bergen H, Cooper J, Turnbull P, Waters K, Ness, J, Kapur N. Suicide following self-harm: findings from the multicentre study of self-harm in England, 2000–2012. J Affect Disorders. 2015;1(175):147–51.
15. Kapur N, Steeg S, Turnbull P, Webb R, Bergen H, Hawton K, et al. Hospital management of suicidal behaviour and subsequent mortality: a prospective cohort study. 2015;2(9):809–16.
16. Conner KR. A call for research on planned vs. unplanned suicidal behavior. Suicide Life-Threat. 2004;34:89–98.
17. Gvion Y, Apter A. Aggression, impulsivity, and suicide behavior: a review of the literature. Arch Suicide Res. 2011;15:93–112.
18. Rimkeviciene J, O'Gorman J, De Leo D. Impulsive suicide attempts: a systematic literature review of definitions, characteristics and risk factors. J Affect Disord. 2015;171:93–104.

04

Reporting of surveillance outcomes and dissemination

Section overview

Disseminating outcomes of surveillance:
Outline of the different levels and formats of reporting and disseminating outcomes generated from surveillance data that can help to impact policy and practice.

Outputs of surveillance systems:
– annual reports outlining the findings of the surveillance system of hospital-presented suicide attempts;
– clinical evidence briefings for use in health-care planning and progress monitoring;
– government briefings to inform health policy and best practice strategy development;
– media briefings;
– periodical reports;
– research papers for peer-reviewed journals.

4.1 Annual reports

STEP 5

Most surveillance systems of hospital-presented suicide attempts and self-harm, whether national or regional, produce annual reports. This is usually a common requirement of the funding organization, such as the Ministry of Health.

Annual reports serve a number of key objectives, such as informing stakeholders about specific trends in suicide attempts and self-harm and describing priorities in terms of (emerging) high-risk groups, frequently used and "new" methods of suicide attempts and self-harm. Another objective of the annual report is to highlight relevant interventions or actions which were guided by the surveillance data on hospital-presented suicide attempts and self-harm. In Ireland, for instance, the number of suicide attempts and cases of self-harm and the rate of repeated acts were taken into account when new specialist self-harm assessment nurses were allocated to hospital emergency departments.

Major stakeholders include government representatives, policy-makers, hospital management and frontline staff (e.g. emergency department nurses, consultant psychiatrists, and first responders such as ambulance staff and police officers). Other relevant stakeholders are mental health and primary care services, general public and media.

In countries with a national suicide prevention strategy, the annual numbers of acts of hospital-presented self-harm (including suicide attempts), rates of self-harm per 100 000

population, and the rates of repeated self-harm, are likely to be used as progress or outcome indicators of the effectiveness of the implementation of the national suicide prevention strategy. For example, the National Strategy for the Reduction of Suicide in Ireland – Connecting for Life, 2015–2020 – has incorporated the National Self-Harm Registry Ireland as a key source of data for the evaluation of the Connecting for Life Strategy (1).

To enhance dissemination of the outcomes of the report among relevant stakeholders, and to increase awareness of the topic of suicide attempts and self-harm, it would be important to plan the publication of the annual report strategically – e.g. by linking the publication of the report to World Suicide Prevention Day, organized by the International Association for Suicide Prevention (IASP) each year on 10 September.

In countries where a surveillance system of hospital-presented suicide attempts and self-harm has been implemented in one or two regions, an annual report which is disseminated among hospitals in regions that are not yet involved could facilitate increased interest among the non-participating hospitals to become part of the surveillance system.

The outline of annual reports is fairly consistent internationally across different surveillance systems of hospital-presented suicide attempts and self-harm. Figure 4.1 shows an example overview of the main themes that could be covered in an annual report.

Figure 4.1. Example overview of annual report

Example overview of annual report
**Foreword**
**Executive summary**
**Suggestions**
**Contribution of the surveillance system**
**Methods**
Background
Definition and terminology
Inclusion criteria
Exclusion criteria
Quality control
Data recording
Data items
Confidentiality
Ethical approval
Registry coverage
Population data
Calculation of rates
A note on small numbers
A note on confidence intervals
**Acknowledgements**
**Section 1 - Hospital presentations**
Self-harm by hospital
Annual change in self-harm presentations to hospital
Variation by month
Variation by day
Variation by the hour
Method of self-harm
Drugs used in overdose
Suggested next care
Self-harm cases discharged from emergency department
Mental health assessment
Repetition of self-harm
**Section 2 - Incidence rates**
Variation by gender and age
Variation by geographical region
Urban and rural district comparison
Rate by city and county
Rate by hospital

4.2
Evidence briefs

STEP 5

Surveillance data on hospital-presented suicide attempts and self-harm can contribute to efficiently updating government representatives, policy-makers and other relevant stakeholders on specific topics in a timely manner using actual or real-time data. Types of evidence briefs include: clinical briefings for use in health-care planning and evaluation, government briefings to inform health policy and best practice strategy development, and media briefings to support appropriate media reporting on cases of suicide, suicide attempts and self-harm.

Ongoing surveillance systems of hospital-presented suicide attempts and self-harm also enable reports to be produced periodically – for instance, on the incidence of hospital-presented suicide attempts and self-harm in the first six months of a year. When the method and scope of data collection (national or subnational) remain the same over time, periodical reports can be used to determine whether there has been an increase or decrease in suicide attempts and self-harm compared to the previous years.

In addition, it would be important to publish regular research bulletins or newsletters which are written for a broader audience including the general public. Such research bulletins or newsletters can focus on a specific topic relating to suicide attempts and self-harm – such as alcohol involvement in hospital-presented suicide attempts and self-harm, aftercare following hospital-presented suicide attempts and self-harm, or trends of repeated suicide attempts and acts of self-harm over time. The National Self-Harm Registry Ireland produces a regular research bulletin [2] .

4.3
Papers for peer reviewed journals

STEP 5

Another major means of dissemination and achieving impact is to publish the outcomes of the surveillance system in peer-reviewed international journals. In addition to annual reports which are mainly used for national purposes, it is important to disseminate the outcomes of national or regional surveillance systems of hospital-presented suicide attempts and self-harm at international level. Peer-reviewed publications strengthen the quality and impact of surveillance systems and enable the surveillance management team to build a track record of specialist knowledge and expertise. This will help to sustain the surveillance system in the long term and will increase the likelihood of obtaining further funding from funding programmes and sources that apply rigorous review criteria.

Examples of relevant papers published in peer review journals can be drawn from the reference list of this chapter (2, 3, 4, 5).

[2] *See:*
http://nsrf.ie/wp-content/uploads/Research%20Bulletins/NSRF%20Research%20Bulletin%20January%202015.pdf, accessed 18 January 2016.

4.4 Informing policy

STEP 5

Outcomes of surveillance systems of suicide attempts and self-harm outlined in annual reports, evidence briefings and peer review papers can be utilized to inform government policy at local and national levels. Examples of contributions that surveillance system data can have are:

- to inform the decision to allocate specialized nurses to emergency departments, prioritizing specific hospitals or emergency care centres on the basis of the volume of suicide attempts and self-harm (6);
- to inform the development of task forces to address the use of specific substances (e.g. medications, pesticides) on the basis of observed patterns of intentional self-poisoning (6);
- to inform the planning and implementation of treatment programmes for individuals who present to hospital after a suicide attempt or act of self-harm;
- to inform the development, implementation and evaluation of national suicide prevention strategies (1, 7).

4.5 References

1. National Office for Suicide Prevention. Connecting for life: Ireland's national strategy to reduce suicide 2015-2020. Dublin: Department of Health; 2015.
2. Arensman E, Larkin C, Corcoran P, Reulbach U, Perry IJ. Factors associated with self-cutting as a method of self-harm: findings from the Irish National Registry of Deliberate Self-Harm. Eur J Public Health. 2014 24 292–7.
3. Bergen H, Hawton K, Waters K, Cooper J, Kapur N. Epidemiology and trends in non-fatal self-harm in three centres in England: 2000–2007. Br J Psychiat. 2010;197:493–8.
4. Ness J, Hawton K, Bergen H, Cooper J, Steeg S, Kapur N, et al. Alcohol use and misuse, self-harm and subsequent mortality: an epidemiological and longitudinal study from the multicentre study of self-harm in England. Emerg Med J. 2015;32:793–9.
5. Perry I.J, Corcoran P, Fitzgerald AP, Keeley HS, Reulbach U, Arensman E. The incidence and repetition of hospital-treated deliberate self-harm: findings from the world's first national registry. PloS One. 2012;7:e31663.
6. Griffin E, Arensman E, Corcoran P, Dillon C, Williamson E, Perry IJ. National Self-Harm Registry Ireland Annual Report 2014. Cork: National Registry of Deliberate Self-Harm; 2015.
7. Crosby A, Ortega L, Melanson C. Self-directed violence surveillance: uniform definitions and recommended data elements, Version 1.0. Atlanta (GA): Centers for Disease Control and Prevention, National Center for Injury Prevention and Control; 2011.

05

Maintenance and sustainability over time

Section overview

Quality and standardization of data collection
Focus on how to maintain standardized data collection and how to sustain suicide attempt and self-harm surveillance systems over time.

In particular:
– the need for quality assurance and quality control;
– the importance of long-term funding for overall sustainability;
– the value and health policy implications of long-term data collection;
– challenges to sustainability and ways to overcome them.

5.1 Maintaining standardization and quality of data collection

STEP 5

In order to maintain the standardization and high quality of the data, it is important that the staff of the surveillance team receive regular supervision and that regular meetings are organized for the data collectors (see Section 3).

All data collectors require standard in-depth training in the data collection methods and procedures, and should be required to attend regular update meetings which review case definitions and related quality control issues. These meetings will be organized by the local or national surveillance management team.

It is suggested that quality control exercises should be conducted at least once every two years, whereby a pair of data registration officers each independently collects data from two hospitals for the same consecutive series of emergency department attendances. It would be important to report the level of agreement between the data registration officers in terms of case ascertainment (Kappa statistics) in annual reports and peer-reviewed papers.

It is also suggested to arrange for an independent review of the surveillance system, to be carried out by independent national or international reviewers after a certain period – such as after the first 3–5 years – and to address any suggestions for enhancing the quality and efficiency of the system.

5.2 Sustainability over time

STEP 5

In order to pursue the key objectives of a surveillance system for hospital-presented suicide attempts and self-harm, long-term sustainability is crucial. For instance, identifying suicide-attempt or self-harm patients with a risk of long-term repetition, and their characteristics, calls for obtaining data on consecutive cases of hospital-presented suicide attempts and self-harm over at least several years. Since attempted suicide and self-harm are associated with high levels of morbidity and mortality by suicide and other causes of premature death, sustaining suicide attempt and self-harm surveillance as a routine health information system should be a key priority. This priority should be endorsed and supported financially by national governments and ministries of health, and should ideally be incorporated into a national suicide prevention strategy or integrated into other surveillance efforts (e.g. on mental health, noncommunicable diseases or injuries).

In most countries, annual suicide mortality data from national statistics offices are published 2–3 years after the relevant year. Consequently, access to real-time surveillance data on hospital-presented suicide attempts and self-harm may be helpful in informing interventions and in their evaluation. In view of the consistency between trends in suicide attempts, self-harm and suicide among males, as observed in Ireland and other countries, data on suicide attempts and self-harm in males can be used as a proxy for suicide. This is a major advantage of these data and represents a convincing argument for funders and policy-makers in the area of suicide prevention. In countries with large geographical areas, where support and funding are available to expand the suicide attempt and self-harm surveillance system but where regular meetings with the surveillance management team are not feasible, virtual meetings can be organized and wider implementation and sustainability of the surveillance system can be achieved by using a "train-the-trainer" model.

One can be proactive in optimizing the use of the information obtained from the suicide attempt and self-harm surveillance systems. For instance, evidence briefs can be prepared for government representatives and others involved in policy development on topics in the media, such as emerging new methods of attempted suicide and self-harm, or clustering of suicide attempts and self-harm (particularly when media reporting cannot be substantiated by evidence and rectification of facts can be provided). This will contribute to improved understanding of the outputs and the importance of sustainability of long-term surveillance of suicide attempts and self-harm.

06

Terminology

Section overview

Review of the terms used for suicide, suicide attempts and self-harm:
– the importance of consensus with regard to the definition used;
– acknowledgement of existing disagreement regarding terms used to describe suicidal behaviours;
– the rationale for the definition used is important for international research, while terms may vary.

6.1 Introduction

Consensus on terms, definitions and classification is important for any surveillance systems and yields benefits in enabling the sharing of coherent surveillance information internationally. Consistency in terminology, definitions and classification facilitates comparison of the outcomes of surveillance systems on suicide attempts and self-harm at global level in terms of comparison of trends, risk groups and patterns of aftercare. This in turn will facilitate international collaboration with regard to building an evidence base for interventions, prevention programmes and best practices for people at risk of and engaging in suicide attempts and self-harm.

The history of terms and definitions used to date for self-harm and suicide attempts are explored in this section.

6.2 A brief history of terms

Research into the epidemiology and etiology of suicidal behaviour is hampered by the lack of agreement on terminology and definitions. For example, over the years a wide range of different terms have been used to indicate varying types of intentional self-harming behaviour with varying degrees of suicidal intent and varying underlying motives – such as *"self-injury"*, *"parasuicide"*, *"attempted suicide"*, *"deliberate self-harm"* and *"self-harm"* (Figure 7.1). Reaching agreement on terminology and definition is further complicated by the varying levels of suicidal intent and heterogeneity of motives reported by people engaging in self-harming behaviour (1).

In 1985, the WHO European regional multicentre study was launched to monitor and to examine non-fatal suicidal behaviour. Researchers from 15 countries agreed that one of the main obstacles to epidemiological research in this area was the lack of a common nomenclature. They agreed that there was a need for terms and definitions of non-fatal suicidal acts that were reasonable from a theoretical point of view and at the same time could be used and applied to surveillance of non-fatal suicidal behaviour (2). In line with Kreitman (3), the WHO European multicentre study group used *"parasuicide"* as an

umbrella term that covered terms such as *"attempted suicide"*, *"deliberate self-harm"* and *"self-poisoning"*. However, as the study expanded to more countries, the term *"parasuicide"* was found to be problematic in practice as it was difficult to translate in some cultures; for instance, *"para"* could be interpreted as "resembling" in some languages or as "mimicking" or "pretending" in others (4), leading to confusion with regard to the meaning of the behaviour involved and the potential for bias. Further, the term *"parasuicide"* was frequently used interchangeably with *"suicide attempt"* demonstrating a difficulty once again with the concept of intent that this term was not helping to clarify (5).

By 1994, *"attempted suicide"* had replaced *"parasuicide"* as the term used in the WHO European multicentre study and was formally replaced in 1999. The use of the terms *"fatal suicidal behaviour"* and *"non-fatal suicidal behaviour"* were also proposed at this point as a way of recognizing the *"suicidal spectrum"*, as it were, while at the same time acknowledging that intent to die might not necessarily be present (6).

However, even in relation to hospital data records, the use of the term *"attempted suicide"* was fluid and the term could mean very different things to different people, as illustrated by the scenario in Figure 6.1 (7).

Figure 6.1.
Terms used to describe intentional self-harming behaviour

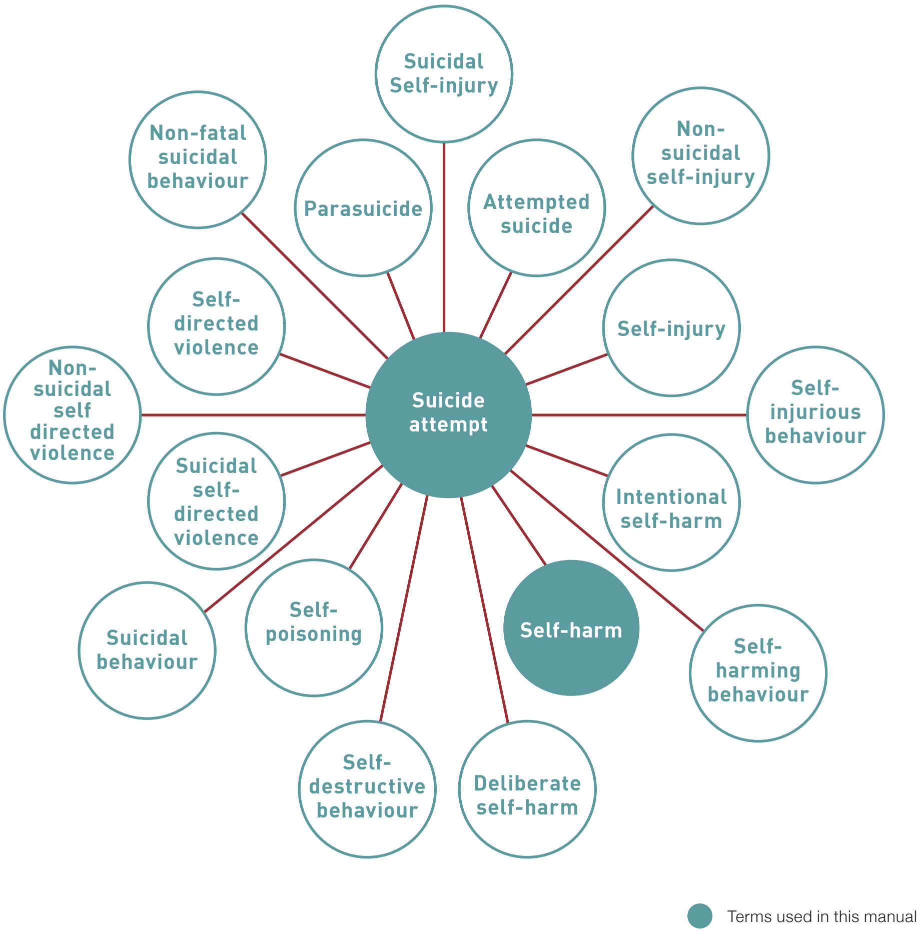

The complexity in deciding on the most suitable term is related to intent to self-harm, which to some extent has to be present in order to bring the act out of the sphere of an accidental occurrence. Consequently, a very fine line has to be followed, where suicidal intent is recognized as fundamental to naming an act as non-accidental yet the term cannot carry the sense of suicidal intent because this also involves a wide range of other motives, such as relief from unbearable thoughts, self-punishment and seeking attention (8, 9, 10).

This was echoed by Silverman et al. (11, 12), who suggested that quantification of suicidal intent was beyond the nomenclature but that it may be more suitable for classification. However, in reworking the O'Carroll (7) nomenclature with a view to removing the either/or dichotomy of suicidal intent, three categories were proposed instead, namely: *"no intent"*, *"uncertain intent"* and *"intent"* (11).

In 2011 in the USA, the Centers for Disease Control and Prevention (CDC) advised on which terms and definitions were recommended in order to ensure consistency in surveillance terms (13). According to the CDC, *"Self-directed violence (SDV) encompasses a range of violent behaviors, including acts of fatal and nonfatal suicidal behavior, and non-suicidal intentional self-harm (i.e., behaviors where the intention is not to kill oneself, as in self-mutilation)"* (13).

In terms of the terminology used in this manual, *"self-harm"* offers a common ground internationally. The concept of self-harm covers a spectrum that ranges in intent and motivation (1). However, it is recognized that this cannot always be translated with the same meaning in other languages and the term *"suicide attempt"* might be preferred in such instances.

Research among young people engaging in self-harm revealed that the majority reported multiple and often contradictory motives underlying their behaviour. The majority also reported both death-oriented and non-death motives at the time of their self-harm act, which reflects ambivalence rather than a static condition (10). What this means in practice is that the priority at the assessment stage is to determine the degree of suicidal intent in those who self-harm, rather than to distinguish between those with and without suicidal intent.

For this reason, terminology such as *"non-suicidal self-injury"* (NSSI), recently introduced as a diagnostic category in the *Diagnostic and Statistical Manual of Mental Disorders (DSM-5)*, may be an appropriate concept within clinical practice but this may not represent appropriate terminology for surveillance (1). As evidence shows suicidal intent to be a fluid rather than a dichotomous "either/or" concept, any descriptor term that takes a side in the either/or argument by excluding or including ideas of intent becomes problematic because it has the potential to exclude a section of the self-harm population (14, 15).

"Self-harm" or *"suicide attempt"* seem appropriate to be used for surveillance because these terms acknowledge and encompass recent findings on self-harm behaviour, namely:

- Subgroups of self-harm patients, characterized as mild versus those with severe self-harm, represent opposite poles of dimensional severity. However, those characterized as mild do not show zero suicidal intent nor do they show absence of suicidal preoccupation.

- Even though research into self-harm subgroups has identified statistically significant and clinically meaningful differences, consistent evidence on homogenous typologies of self-harm is lacking and the dimensionality of self-harm severity reflects the complexity of self-harm (1).

At the global level, terminology should be approached with a certain amount of flexibility, taking into account varying cultural contexts and the fact that different countries may choose terms that translate more accurately in their languages.

For clarity and uniformity of research, and agreement on inclusion and exclusion criteria of cases, the definition used needs to be consistent.

6.3 References

1. Arensman E, Keeley H. Defining intent. Psychiatry Professional. 2012;Spring:8–9 (http://nsrf.ie/wp-content/uploads/journals/12/ArensmanKeeley(2012).pdf, accessed 18 January 2016).
2. Bille-Brahe U, Schmidtke A, De Leo D. 2004. The WHO/EURO multi-centre study on suicidal behaviour; it's background, history, aims and design. In: De Leo D, Bille-Brahe U, Kerkhof A, Schmidtke A, editors. Suicidal behaviour: theories and research findings. Göttingen: Hogrefe & Huber Publishers; 2004:17–39.
3. Kreitman N. Parasuicide. London: John Wiley; 1977.
4. De Leo D, Burgis S, Bertolote J, Kerkhof A, Bille-Brahe U. Definitions of suicidal behaviour. In: De Leo D, Bille-Brahe U, Kerkhof A, Schmidtke A, editors. Suicidal behaviour: theories and research findings. Göttingen: Hogrefe & Huber Publishers; 2004:17-39.
5. Bille-Brahe U, Schmidtke A, Kerkhof AJ, De Leo D, Lönnqvist J, Platt S, et al. Background and introduction to the WHO/EURO multicentre study on parasuicide. Crisis. 1995;16:72–78, 84.
6. De Leo D, Bertolote JM, Schmidtke A, Bille-Brahe U, Kerkhof A. Definitions in suicidology: the evidence-based and the public health approach. In: Proceedings of the 20th World Congress of the International Association for Suicide Prevention, Athens, 1999:194.
7. O'Carroll PW, Berman AL, Maris RW, Moscicki EK, Tanney BL, Silverman MM.. Beyond the Tower of Babel: a nomenclature for suicidology. Suicide Life Threat Behav. 1996;26:237–52.
8. Hjelmeland H, Hawton K, Nordvik H, Bille-Brahe U, De Leo D, Fekete S, et al. Why people engage in parasuicide: a cross-cultural study of intentions. Suicide Life Threat Behav. 2002;32:380–93.
9. McAuliffe C, Arensman E, Keeley HS, Corcoran P, Fitzgerald AP. Motives and suicide intent underlying hospital treated deliberate self-harm and their association with repetition. Suicide Life Threat Behav. 2007;37:397–408.
10. Scoliers G, Portzky G, Madge N, Hewitt A, Hawton K, de Wilde EJ. et al. Reasons for adolescent deliberate self-harm: a cry of pain and/or a cry for help? Findings from the child and adolescent self-harm in Europe (CASE) study. Soc Psychiatry Psychiatr Epidemiol. 2009;44:601–7.
11. Silverman MM, Berman AL, Sanddal ND, O'Carroll PW, Joiner TE. Rebuilding the Tower of Babel: a revised nomenclature for the study of suicide and suicidal behaviors. Part 1: Background, rationale, and methodology. Suicide Life Threat Behav. 2007;37:248–63.
12. Silverman MM, Berman AL, Sanddal ND, O'Carroll PW, Joiner TE. Rebuilding the Tower of Babel: a revised nomenclature for the study of suicide and suicidal behaviors. Part 2: Suicide-related ideations, communications, and behaviors. Suicide Life Threat Behav. 2007;37:264–77.
13. Crosby A, Ortega L, Melanson C. Self-directed violence surveillance: uniform definitions and recommended data elements, Version 1.0. Atlanta (GA): Centers for Disease Control and Prevention, National Center for Injury Prevention and Control; 2011.
14. Jobes DA, David M, Overholser JC, Joiner TE Jr. Ethical and competent care of suicidal patients: contemporary challenges, new developments, and considerations for clinical practice. Prof Psychol Res Pract. 2008;39:405–13.
15. Rudd MD. What's in a name …. . Suicide Life Threat Behav. 1997;27:326–7.

07

Overview of existing surveillance systems or projects for suicide attempts and self-harm

Section overview

Review of overall literature:

– to provide examples of surveillance systems at national or subnational level;

– to demonstrate the use of representative catchment populations;

– to demonstrate the usefulness of surveillance in low- and middle-income countries.

Best practice findings:

- Use of a minimal data set is found to be most effective for surveillance of large populations.
- Long-term surveillance is preferable.
- Where long-term surveillance is problematic, conducting time-limited, single or multisite surveillance projects provides valuable data.

7.1 Background

As outlined in the CDC Vision for Public Health Surveillance in the 21st Century (1), any surveillance system has to address both national and global objectives, namely:

- For the world: a globally connected network of public health surveillance systems that optimizes disease prevention and health promotion.
- For each nation: a fully functioning, efficient set of national public health surveillance systems that protect the nation's public health and provide timely information to guide public health action challenges.

These fundamental objectives of surveillance are pursued by this manual.

7.2 Surveillance of suicide attempts and self-harm across the world

In 2013, the International Association for Suicide Prevention (IASP) and the WHO Department of Mental Health and Substance Abuse initiated the IASP-WHO global survey on suicide prevention to ascertain what information exists on national suicide prevention strategies, including surveillance of suicide and suicide attempts, by writing primarily to IASP members in countries. Ninety of the 157 countries which received the survey questionnaire responded – a 57% response rate. With regard to the question as to whether a registry of any kind for suicide attempts was present, only 18% (16 out of 89 countries) confirmed the presence of such a registry, with countries in the WHO Region of the Americas and the European Region being overrepresented.

For the purpose of this manual, a preliminary review was conducted to examine surveillance systems or projects worldwide. The review does not purport to be exhaustive (many efforts remain unpublished), but rather aims to provide an overview of surveillance systems or projects in both high-income and low- and middle-income countries (see Annex 3 for an overview).

In recent years, surveillance of suicide attempts and self-harm has been increasingly prioritized at national and international levels and uses different methods: dedicated registries, multicentre projects, national/international statistics and databases, and individual studies (Figure 7.1).

Figure 7.1. Different methods used in surveillance of hospital-presented suicide attempts and self-harm

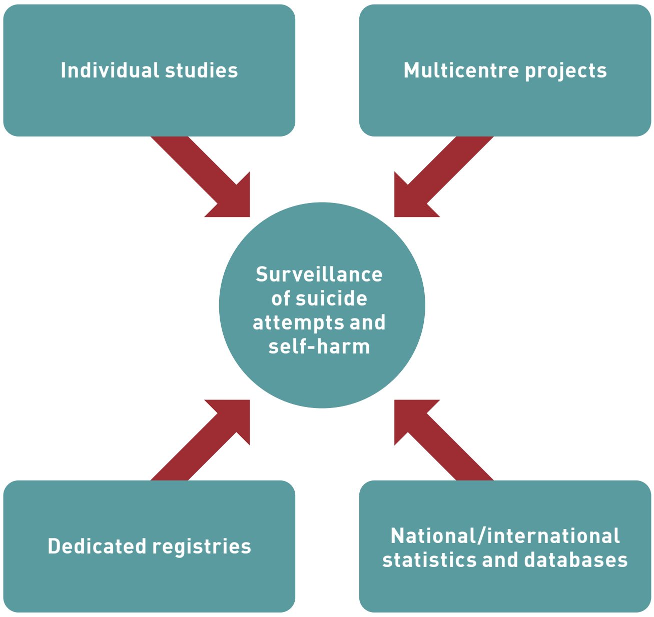

7.2.1 Dedicated registries for hospital-presented suicide attempts and self-harm

Dedicated registries focus solely on the collection of data related to hospital-presented suicide attempts and self-harm. Data collectors are recruited and trained for the purpose of obtaining data on consecutive hospital presentations resulting from attempted suicide and self-harm. Surveillance is conducted on the full population for a specific geographical area or a whole country.

In establishing a surveillance system, the advantage of a dedicated registry is that service provision, resource deployment and guidelines/protocols for self-harm management are very specific to that area. Real-time data can be gathered and used to guide service delivery, particularly the assessment and management of hospital-presented suicide attempts and self-harm.

The National Self-Harm Registry Ireland is currently the only registry at national level covering consecutive cases of self-harm (including suicide attempts) presenting to all general hospitals in the country (2, 3). However, at subnational level, the Self-Harm Registry in Northern Ireland, which was developed on the basis of the registry in Ireland, and the Registry of Suicide Attempts in Flanders, Belgium, cover all hospital-presented suicide attempts and self-harm acts in their respective regions (4).

The longest established registry is the Oxford Monitoring System of Deliberate Self-harm, which was established in 1976 and collects all information on self-harm (including suicide attempt) cases presenting to John Radcliffe Hospital, Oxford, United Kingdom. It is unique in Europe in terms of the completeness of data obtained and its duration. Based on Oxford's longstanding experience and in order to serve as a resource for others intending to set up a self-harm surveillance system, the procedures used for monitoring, case definition and identification, investigation of repetition, data protection and ethical issues have been outlined (5). The Oxford monitoring system provided the template for the United Kingdom's Multicentre Monitoring System of Deliberate Self-Harm which was established in 2000 and covers consecutive hospital presentations of self-harm in the cities of Derby, Manchester and Oxford (6).

The registries are fairly consistent with regard to definition of the target behaviour, inclusion criteria, standard operating procedures and provision of training for officers and researchers involved in data collection. Findings from these registries suggest a correlation of figures with prevention initiatives and an influence of the registries on national strategies and legislation.

7.2.2 Multicentre projects

Multicentre projects pool data to arrive at wider epidemiological profiles of persons engaging in suicide attempts and self-harm, and to compare incidence rates between geographical areas and countries. Data on suicide attempts and self-harm are usually drawn from general hospital records, registries and vital statistics.

In line with the implementation of registries internationally, in recent years multicentre projects on self-harm and suicide attempts have expanded from high-income to low- and middle-income countries (7).

The WHO European regional multicentre study on suicidal behaviours has been conducted over the longest time period (1989–2005) and has covered the largest number of geographical areas in European countries (8).

Based on the experiences of the WHO European regional multicentre study, and in cooperation with the WHO European Regional Network on Suicide Research and Prevention, the MONitoring SUicidal behaviour in Europe study project (MONSUE) was established in 2006. The aim of the study, which involved nine European countries, was to investigate hospital-presented suicide attempts based on intent and lethality. Two subgroups were defined – one on non-serious suicidal behaviour (NSSB) including those whose intent to die was considered minimal or absent, and one on serious suicidal behaviour (SSB). A further aim was to determine differences, if any, between these subgroups (9).

The WHO Western Pacific regional study on Suicide Trends in At-Risk Territories (START) is an international multisite initiative, established in 2001, that aims to stimulate suicide research and prevention in the WHO Western Pacific Region. The central component of the study is the development of registration systems for fatal and non-fatal suicidal behaviours (7).

The Council of Ministers of Health of Central America (COMISCA) has mandated to improve public health in this region by joining forces, combining resources and agreeing on a collective public health blueprint. COMISCA is the political body of the Central American Integration System (SICA), which aims to identify and prioritize regional health problems and is composed of each of the ministers of health of eight countries (Belize, Costa Rica, Dominican Republic, El Salvador, Guatemala, Honduras, Nicaragua, Panama). CDC is one of COMISCA's main partners.

WHO's Suicide Prevention - Multisite Intervention Study on Suicidal Behaviours (SUPRE-MISS) was launched in 2000 to examine suicidal behaviours and interventions at culturally diverse sites. A community survey and hospital-based case registration of suicide attempters for the clinical trial component of the study provided very valuable results (10).

Multicentre projects provide an epidemiological overview of wider trends and provide good practice frameworks and information on the planning, development and maintenance of multisite surveillance systems. Findings from these projects have provided new knowledge (e.g. importance of self-poisoning by pesticides as a method, identification of different levels of intent) and have informed research and national suicide prevention strategies.

7.2.3 National/international statistics and databases

In a number of countries (e.g. Australia, Belgium, Canada, Denmark, France, New Zealand, Norway, Sweden, United Kingdom, USA) existing health information systems and databases include hospital data on suicide attempts and self-harm as part of a wider brief of collecting national or nationally representative data on injuries and poisonings.

In countries, where resources are scarce, effective use of hospital data can provide valuable information on the prevalence of medically treated suicide attempts and self-harm acts.

The USA is an example of a country with a large population that combines various surveillance systems to provide a representative sample of hospital-presented suicide attempts.

In Europe, there is one relevant international database which records data on medically treated suicide attempts: the European Injury Database (IDB). The IDB is a unique data source that contains standardized cross-national data on the external causes and circumstances of injuries treated in emergency departments. The first results of the IDB "intentional self-harm module" in 2013, which looked at self-injury and suicide attempts in European countries, demonstrate that this tool has value in providing a broad overview of trends in hospital-presented suicide attempts and self-harm (11).

National databases vary in terms of sampling and data collection procedures for hospital-presented suicide attempts and self-harm, and this may limit comparison of outcomes across countries. However, each country uses ICD coding in classifying intentional self-harm, sometimes in addition to other classifications. The existing national databases provide the potential for morbidity data to be given equal importance in monitoring as that given to mortality data.

7.2.4 Individual studies

Individual studies conduct registration of hospital-presented suicide attempts and self-harm for a time-limited period in a specific geographical area or on a specific population (e.g. a city, a particular ethnic group, or people with a specific disorder). While ongoing surveillance is preferable, individual studies provide insight into the prevalence and incidence of suicide attempts, self-harm and associated factors which can contribute to the identification of further areas for research, development or intervention. Individual studies may also form the basis for developing a surveillance system of hospital-presented suicide attempts and self-harm. Increasing numbers of studies are found in low- and middle-income countries.

7.3 References

1. CDC's Vision for Public Health Surveillance in the 21st Century. Morb Mortal Wkly Rep. 2012;61(Suppl):1–40.
2. Griffin E, Arensman E, Corcoran P, Dillon C, Williamson E, Perry IJ. National Self-Harm Registry Ireland Annual Report 2014. Cork: National Registry of Deliberate Self-Harm; 2015.
3. Perry IJ, Corcoran P, Fitzgerald AP, Keeley HS, Reulbach U, Arensman E. The incidence and repetition of hospital-treated deliberate self-harm: findings from the world's first national registry. PloS One. 2012;7:e31663.
4. Vancayseele N, De Jaegere E, Portzky G, van Heeringen C. De epidemiologie van suïcide-pogingen in Groot Gent (The epidemiology of suicide attempts in bigger Gent) (Jaarverslag 2011). Ghent: Eenheid voor Zelfmoordonderzoek; 2012.
5. Hawton K, Bale L, Casey D, Shepherd A, Simkin S, Harriss L. Monitoring deliberate self-harm presentations to general hospitals. Crisis. 2006;27:157–63.
6. Hawton K, Bergen H, Casey D, Simkin S, Palmer B, Cooper J, et al. Self-harm in England: a tale of three cities. Multicentre study of self-harm. Soc Psych Psychiatr Epidem. 2007;42:513–21.
7. De Leo D, Milner A, Fleischmann A, Bertolote J, Collings S, Amadeo S, et al. The WHO START study: suicidal behaviors across different areas of the world. Crisis. 2013;34:156–63.
8. Schmidtke A, Bille-Brahe U, DeLeo D, Kerkhof A. Suicidal behaviour in Europe: results from the WHO/EURO Multicentre Study on Suicidal Behaviour. Göttingen: Hogrefe Publishing; 02. 2004.
9. Carli V, Mandelli L, Zaninotto L, Iosue M, Hadlaczky G, Wasserman D, et al. Serious suicidal behaviors: socio-demographic and clinical features in a multinational, multicenter sample. Nord J Psychiatr. 2014;68:44–52.
10. Fleischmann A, Bertolote JM, De Leo D, Botega N, Phillips M, Sisask M, et al. Characteristics of attempted suicides seen in emergency-care settings of general hospitals in eight low- and middle-income countries. Psychol Med. 2005;35:1467–74.
11. Injuries in the European Union, Report on injury statistics 2008–2010. Amsterdam: Eurosafe; 2013.

08

Relevant reading

Antretter E, Dunkel D, Haring C, Corcoran P, De Leo D, Fekete S, et al. The factorial structure of the Suicide Intent Scale: a comparative study in clinical samples from 11 European regions. Int J Psychiatr Res. 2008;17:63–79.

Arensman E, Larkin C, Corcoran P, Reulbach U, Perry IJ. Factors associated with self-cutting as a method of self-harm: findings from the Irish National Registry of Deliberate Self-Harm. Eur J Public Health. 2014;24:292–7.

Carroll R, Metcalfe C, Gunnell D. Hospital presenting self-harm and risk of fatal and non-fatal repetition: systematic review and meta-analysis. PLoS One. 2014;9:e89944.

Crosby A, Ortega L, Melanson C. Self-directed violence surveillance: uniform definitions and recommended data elements, Version 1.0. Atlanta (GA): Centers for Disease Control and Prevention, National Center for Injury Prevention and Control; 2011.

De Leo D, Burgis S, Bertolote JM, Kerkhof AJ, Bille-Brahe, U. Definitions of suicidal behavior: lessons learned from the WHO/EURO multicentre study. Crisis. 2006;27(1):4–15.

De Leo D, Milner A, Fleischmann A, Bertolote J, Collings S, Amadeo S, et al. The WHO START study: suicidal behaviors across different areas of the world. Crisis. 2013;34:156–63.

Fleischmann A. Effectiveness of brief intervention and contact for suicide attempters: a randomized controlled trial in five countries. Bull World Health Organ. 2008;86:703–9.

Fleischmann A, Bertolote JM, De Leo D, Botega N, Phillips M, Sisask M, et al. Characteristics of attempted suicides seen in emergency-care settings of general hospitals in eight low- and middle-income countries. Psychol Med. 2005;35:1467–74.

Griffin E, Arensman E, Corcoran P, Dillon C, Williamson E, Perry IJ. National Self-Harm Registry Ireland Annual Report 2014. Cork: National Registry of Deliberate Self-Harm; 2015.

Hawton K, Bergen H, Cooper J, Turnbull P, Waters K, Ness J, Kapur N. Suicide following self-harm: findings from the multicentre study of self-harm in England, 2000–2012. J Affect Disorders. 2015;1:147–51.

Hawton K, Bergen H, Kapur N, Cooper J, Steeg S, Ness J, Waters K. Repetition of self-harm and suicide following self-harm in children and adolescents: findings from the Multicentre Study of Self-harm in England. J Child Psychol Psychiatr. 2012;53:1212–9.

Hawton K, Bergen H, Casey D, Simkin S, Palmer B, Cooper J, et al. Self-harm in England: a tale of three cities. Multicentre study of self-harm. Soc Psych Psychiatr Epidem. 2007;42:513–21.

Jiang C, Li X, Phillips MR, Xu Y. Matched case-control study of medically serious attempted suicides in rural China. Shanghai Arch Psychiatr. 2013;25:22–31.

Kapur N, Steeg S, Turnbull P, Webb R, Bergen H, Hawton K, et al. Hospital management of suicidal behaviour and subsequent mortality: a prospective cohort study. Lancet Psychiatry. 2015;2:809–16.

Lilley R, Owens D, Horrocks J, House A, Noble R, Bergen H, et al. Hospital care and repetition following self-harm: multicentre comparison of self-poisoning and self-injury. Br J Psychiatry J Ment Sci. 2008;192:440–5.

Perry IJ, Corcoran P, Fitzgerald AP, Keeley HS, Reulbach U, Arensman E. The incidence and repetition of hospital-treated deliberate self-harm: findings from the world's first national registry. PloS One. 2012;7:e31663.

Platt S, Bille-Brahe U, Kerkhof A, Schmidtke A, Bjerke T, Crepet P, et al. Parasuicide in Europe: the WHO/EURO multicentre study on parasuicide. I. Introduction and preliminary analysis for 1989. Acta Psychiatr Scand. 1992;85:97–104.

Posner K, Oquendo MA, Gould M, Stanley B, Davies M. Columbia Classification Algorithm of Suicide Assessment (C-CASA): classification of suicidal events in the FDA's pediatric suicidal risk analysis of antidepressants. Am J Psychiatry. 2007;164:1035–43.

Rajendra R, Krishna M, Majgi S, Heggere N, Robinson C, Poole R. A feasibility study to establish a deliberate self-harm register in a state hospital in southern India. Br J Med Pract. 2015;8:807.

Silverman MM, Berman AL, Sanddal ND, O'Carroll PW, Joiner TE. Rebuilding the Tower of Babel: a revised nomenclature for the study of suicide and suicidal behaviors. Part 1: Background, rationale, and methodology. Suicide Life Threat Behav. 2007;37:248–63.

Silverman MM, Berman AL, Sanddal ND, O'Carroll PW, Joiner TE. Rebuilding the Tower of Babel: a revised nomenclature for the study of suicide and suicidal behaviors. Part 2: Suicide-related ideations, communications, and behaviors. Suicide Life Threat Behav. 2007;37:264–77.

Preventing suicide: a global imperative. Geneva: World Health Organization; 2014.

Preventing suicide: a resource for non-fatal suicidal behaviour case registration. Geneva: World Health Organization; 2014.

Mental Health Action Plan 2013–2020. Geneva: World Health Organization; 2013.

Xu Y, Phillips MR, Wang L, Chen Q, Li C, Wu X. Retrospective identification of episodes of deliberate self-harm from emergency room registers in general hospitals: an example from Shanghai. Arch Suicide Res. 2013;17:345–59.

Annex 1.
Example of an information leaflet describing a surveillance system for suicide attempts and self-harm

Front of leaflet

The National Suicide Research Foundation

- is the principal Irish centre for research on suicidal behaviour
- carries out a wide range of research studies in addition to operating the National Self-Harm Registry Ireland
- is registered with the Data Protection Agency and complies with Irish data protection legislation
- was granted ethical approval for the operation of the Registry by national and local ethics committees
- produces an annual report of the findings of the National Self-Harm Registry Ireland which is available on our website or by contacting our offices

The Registry is funded by the Health Service Executive's National Office for Suicide Prevention.

Feidhmeannacht na Seirbhíse Sláinte
Health Service Executive

The Registry

This hospital is providing data to the National Self-Harm Registry Ireland

All cases of self-harm presenting to the emergency department are recorded in a way that preserves patient anonymity and confidentiality

This leaflet provides more information on what is meant by self-harm, why the data are being recorded and how they will be used

If you would like any more information about the Registry, please contact us at the NSRF

Where to find help

Aware (1890 303 302): helps people and families affected by depression

Childline (1800 666 666): helps young people in distress

Console (1800 201 890): provides support and information for those affected by suicide

Pieta House (01 601 00 00): centre for the prevention of self-harm

Samaritans (116 123): provides emotional support 24 hours a day to people in distress

NATIONAL SELF-HARM REGISTRY IRELAND

NSRF

National Suicide Research Foundation

4.28 Western Gateway Building
University College Cork

Tel: 021 420 5551
email: info@nsrf.ie
website: www.nsrf.ie

Back of leaflet

What is the National Self-Harm Registry Ireland?

It is a system of recording all self-harm presentations made to hospital emergency departments in Ireland

The Registry is an integral part of the Irish national suicide prevention strategy and forms the basis for improved services for those who are at high risk of suicidal behaviour

The Registry was established in 2000 by the National Suicide Research Foundation at the request of the Department of Health and Children

The Registry is funded by the Health Service Executive's National Office for Suicide Prevention

The Registry operates under the direction of Professor Ivan J Perry, who is also Head of the Department of Epidemiology and Public Health at University College, Cork

The aims of the National Self-Harm Registry Ireland are:

- to establish the extent and nature of hospital-treated self-harm in Ireland
- to monitor trends over time and by area
- to contribute to policy development in the area of suicidal behaviour
- to help the progress of research and prevention

What is self-harm?

- It is a non-fatal act
- It is deliberately initiated
- The individual knows it may cause physical harm to her or himself and may cause death
- It includes suicide attempts but there may be other intentions involved

Some findings of the National Self-Harm Registry Ireland:

- In 2014, approximately 11,000 self-harm presentations were made to hospital emergency departments in Ireland
- These presentations were made by approximately 8,500 individuals, as some people made multiple presentations to hospital
- The Irish rate of self-harm is higher in women than in men and the highest rate is among young people
- Almost half of all Irish self-harm presentations are by people aged under 30 years
- Three in every four self-harm presentations are the result of an intentional drug overdose
- Alcohol is involved in about 40% of self-harm episodes.

Source: National Self-Harm Registry Ireland. Reprinted by permission of the National Suicide Research Foundation, Cork, Ireland.

Annex 2. Example of a paper data registration form

**Data collector**	_ _ _
**Date of registration**	_ _ Day / _ _ Month / _ _ _ _ Year
**Hospital number**	_ _ _
**Unique event number**	_ _ _ _ _ _ _
**Unique person identification number**	_ _ _ _ _ _ _ _ _ _
**Sex**	_ Male _ Female _ Transsexual
**Date of birth**	_ _ Day / _ _ Month / _ _ _ _ Year
**Age**	_ _ years
**Postal / area code**	_ _ _ _ _
**Date of presentation**	_ _ Day / _ _ Month / _ _ _ _ Year
**Time of presentation**	_ _ Hour / _ _ Minute
**Mode of arrival at the hospital**	_ Ambulance _ Fire brigade _ Police _ Coast guard<br>_ Self-presentation _ Other (specify) _ _ _
**Seen by on arrival at the hospital**	_ Emergency department doctor _ Nurse<br>_ Other hospital staff (specify) _ _ _
**Date of the self-harm**	_ _ Day / _ _ Month / _ _ _ _ Year
**Day of the week of the self-harm**	_ _ _
**Time of the self-harm**	_ _ Hour / _ _ Minute
**Location of the self-harm**	_ _ _
**Method(s) according to ICD-10 codes**	_ _ _ _ _ _ _ _ _ _ _ _<br>(if applicable, specify substance used and quantity _ _ _)
**Medical severity of the self-harm**	_ Treatment in specialized unit _ Surgery under anaesthesia<br>_ Extensive medical treatment _ Evidence of altered level of consciousness _ Unconscious at or prior to presentation
**Statement of intention to die**	_ No _ Yes (describe) _ _ _
**History of self-harm (previous self-harm)**	_ No _ Yes (describe) _ _ _
**Psychological / psychiatric assessment in the hospital**	_ No _Yes, by _ Psychiatrist _ Clinical psychologist<br>_ Nurse _ Social worker _ Other (specify) _ _ _
**Diagnosis (any diagnosis associated with the person)**	_ No _ Yes (specify) _ _ _
**Admission to the hospital**	_ No _Yes, to _ Intensive care unit _ Emergency unit<br>_ Psychiatric inpatient unit _ Other (specify) _ _ _
**Discharge from the hospital**	_ _ Day / _ _ Month / _ _ _ _ Year; _ _ Hour / _ _ Minute

Annex 3. Examples of methods used in surveillance of hospital-presented suicide attempts and self-harm

Examples of dedicated registries for hospital-presented suicide attempts and self-harm

Location	Web link	Coverage	Centres	Population	Duration	Source
**Ireland**	http://nsrf.ie/wp-content/uploads/reports/NSRF%20National%20Self-Harm%20Registry%20Ireland%20Annual%20Report%202014.pdf	National (26 counties)	40	All	From 2003 (ongoing)	Hospital-based monitoring (including all hospitals)
**Costa Rica**	https://www.ministeriodesalud.go.cr/index.php/centro-de-prensa/noticias/662-noticias-2014/698-costa-rica-vigila-los-intentos-de-suicidio-por-primera-vez-desde-el-2013	National	-	All	From 2013 (ongoing)	Presentations to public and private hospitals
**Northern Ireland**	http://www.publichealth.hscni.net/publications/northern-ireland-registry-self-harm-annual-report-201314	Subnational	3	All	From 2007 (ongoing)	Presentations to emergency departments
**UK multicentre**	http://cebmh.warne.ox.ac.uk/csr/mcm/	Subnational (Derby, Manchester, Oxford	6	51 206 (sample analysed)	From 2000 (ongoing)	Treated in health system
**UK Oxford**	http://cebmh.warne.ox.ac.uk/csr/monitoring.html	Subnational (Oxford)	1	450 000	From 1976 (ongoing)	Presentations to inpatient and outpatient departments
**Belgium**	http://bjp.rcpsych.org/content/183/3/260	Subnational (Flanders)	62	225 393	From 1986 (ongoing)	General and psychiatric presentations (also prisons and sentinel centres)

Examples of multicentre projects

Name	Countries	Centres	Population	Duration	Source
**WHO European regional multicentre study on suicidal behaviour**	19*	25	6 million	1989–2005	Treated in health system
**WHO European regional study MONSUE (MONitoring SUicidal behaviour in Europe)**	9**	10	2.5 million	2006–2010	Treated in health system
**WHO START (Suicide Trends in At Risk Territories)**	10***	14	106 million	From 2001 (ongoing)	Treated in health system
**COMISCA (Council of Ministers of Health of Central America and the Dominican Republic)**	8****	8	-	From 2010 (ongoing)	Treated in health system
**WHO Suicide Prevention - Multisite Intervention Study on Suicidal Behaviours (SUPRE-MISS)**	8*****	8	2 million	2000–2004	Treated in health system

* Austria, Belgium, Denmark, Estonia, Finland, France, Germany, Greece, Hungary, Ireland, Italy, Netherlands, Norway, Slovenia, Spain, Sweden, Switzerland, Turkey, United Kingdom.

** Belgium, Estonia, Germany, Hungary, Italy, Slovenia, Spain, Sweden, Switzerland.

*** Australia, Brazil, China, Fiji, Italy, Mongolia, New Zealand, Philippines, Tonga, Vanuatu (as well as territories: French Polynesia, Guam, and China, Hong Kong Special Administrative Region).

**** Belize, Costa Rica, Dominican Republic, El Salvador, Guatemala, Honduras, Nicaragua, Panama.

***** Brazil, China, Estonia, India, Iran, South Africa, Sri Lanka, Viet Nam.

Examples of national/international statistics and databases

Location	Name	Web link	Coverage	Source
**Australia**	National Hospital Morbidity Database (NHMD)	http://www.aihw.gov.au/hospitals-data/national-hospital-morbidity-database	National	Almost all public and private hospitals
**Belgium**	Minimum Hospital Data Set (MHD)	http://hspm.org/countries/belgium25062012/livinghit.aspx?Section=2.7%20Health%20information%20management&Type=Section	National	All hospitals
**Belize**	Belize Health Information System (BHIS)	http://health.gov.bz/www/health-projects/health-information-system/bhis-expansion	National	All public and private hospitals
**Canada**	Discharge Abstract Database (DAD)	https://www.cihi.ca/en/types-of-care/hospital-care/acute-care/dad-metadata	National	Acute care facilities (except Quebec)
	Hospital Morbidity Database (HMDB)	https://www.cihi.ca/en/types-of-care/hospital-care/acute-care/hmdb-metadata	National	Acute care facilities (including day surgery facilities in Quebec)
	Ontario Trauma Registry (OTR)	https://www.cihi.ca/en/types-of-care/specialized-services/trauma-and-injuries/ontario-trauma-registry-otr-metadata	Subnational	Trauma facilities
	Hospital Mental Health Database (HMHDB)	https://www.cihi.ca/en/types-of-care/specialized-services/mental-health-and-addictions/hospital-mental-health-database	National	Hospitalizations for mental illness
	Canadian Hospitals Injury Reporting and Prevention Program (CHIRPP)	http://www.phac-aspc.gc.ca/injury-bles/chirpp/index-eng.php	Subnational	10 paediatric and 6 general hospitals
**Denmark**	Danish National Patient Register (DNPR)	http://sjp.sagepub.com/content/39/7_suppl/30.refs	National	All hospitals
	The Register for Suicide Attempts (RSA)	http://www.danmedbul.dk/DMB_2004/0404/0404-artikler/DMB3621.pdf	Subnational (Funen county)	All hospitals
**Dominican Republic**	Sistema Nacional de Vigilancia Epidemiológica (SINAVE)	http://www.epidemiologia.salud.gob.mx/dgae/sinave/intd_sinave.html	National	Public and private health services

Location	Name	Web link	Coverage	Source
**Fiji**	Public Health Information System (PHIS)	http://www.fiji.gov.fj/Media-Center/Press-Releases/HEALTH-DATABASE-TO-STRENGTHEN-HEALTH-SERVICE-DELIV.aspx	National	All government hospitals
**Finland**	Inpatient Health Care	http://www.stat.fi/til/thlaho/index_en.html	National	All hospitals
**France**	Observatoire national du suicide (ONS)	http://www.inpes.sante.fr/10000/themes/sante_mentale/observatoire_national_suicide.asp	National	All public and private hospitals
**New Zealand**	National Minimum Dataset of Discharge Data (NMDS)	http://www.health.govt.nz/nz-health-statistics/national-collections-and-surveys/collections/national-minimum-dataset-hospital-events	National	All public and private hospitals
**Norway**	Norwegian Patient Register (NPR)	https://helsedirektoratet.no/english/norwegian-patient-register	National	All hospitals
**Philippines**	Online Electronic Injury Surveillance System (ONEISS)	http://oneiss.doh.gov.ph/oneiss/login.php	Subnational	Government and private hospitals
**Sweden**	National Patient Registry (NPR)	http://www.jpi-dataproject.eu/Home/Database/361?topicId=1	National	All hospitals
**United Kingdom**	Health & Social Care Information Centre (HSCIC); Injury Observatory Britain & Ireland	http://www.hscic.gov.uk/; http://www.injuryobservatory.net/category/data+crosscut	National	All National Health Service hospitals and community health-care centres
	All Wales Injury Surveillance System (AWISS)	http://www.ncbi.nlm.nih.gov/pmc/articles/PMC1730805	Subnational	All hospitals
**USA**	Web-based Injury Statistics Query and Reporting System (WISQARS)	http://www.cdc.gov/injury/wisqars	45 different federal data systems operated by 16 different agencies and 3 private injury registry systems	Full list of databases: http://www.cdc.gov/injury/wisqars/InventoryInjuryDataSys.html
**European Union**	European Injury database (IDB)	http://ec.europa.eu/health/data_collection/databases/idb/index_en.htm	National	All hospitals

Examples of individual studies

**Afghanistan (Medica Mondiale et al., 2008)**	A report for Medica Mondiale looking at self-immolation in Afghan women analysed data from hospital burn units in the three provinces of Heart, Kabul and Wardak.
**Australia (Milner et al., 2013)**	Study to assess treatment priority given to self-harmers presenting to the emergency department of the Gold Coast Hospital, Queensland, Australia from 2005 to 2010.
**Belgium (De Munck et al., 2009)**	Nine-year monitoring study of suicide attempts in adolescents and young adults in the Accident and Emergency Department of the Gent University, Flanders, Belgium, between January 1996 and December 2004.
**China (Xu et al., 2013)**	The Shanghai Jiading District Hospital Emergency Room Register was used to ascertain retrospectively the prevalence and characteristics of episodes of medically treated deliberate self-harm from 2007 to 2010.
**China (Chen et al., 2009)**	All Nantou residents in China, Province of Taiwan who had carried out self-harm were recorded in the Nantou Suicide Register from July 2000 to February 2003 and then followed up through to December 2005.
**Fiji (Peiris-John et al., 2013)**	As part of the Traffic-Related Injury in the Pacific (TRIP) project, a population-based registry named the Fiji Injury Surveillance in Hospitals System, was established in October 2005 in Viti Levu and ran for 12 months. Data were captured on hospital admissions of 12 hours and longer due to acute injury or poisoning.
**India (Rajendra et al., 2015)**	A feasibility study was conducted on the establishment of a self-harm register in the Mysore Medical College and Research Institution. Data were collected by interview and from records for all individuals presenting with deliberate self-harm over a six-month period in 2012.
**India (Gururaj et al., 2001)**	The study analysed data collected on suicide attempters presenting to three government and nine private hospitals (out of about 120 hospitals) in the city of Bangalore from 1998-1999. All hospitals ran a 24-hour emergency service department.

**Iran (Sharif-Alhoseini M et al., 2012)**	The national injury surveillance system (ISS) was used to identify suicide attempts from 2005 to 2008. The ISS is hospital-based and limited to injured patients who enter the emergency departments of governmental hospitals, which account for 68.9% of the 942 hospitals in Iran. They cover the whole country, their services are cheap and available for all people, and therefore, everyone can use their services.
**Malaysia (Sinniah et al., 2014)**	A review identified a total of 38 studies on suicide attempts in Malaysia. The data were mainly gathered from government hospitals (63%) during admission of suicide patients after a suicide attempt. The remaining were retrospective data (34%) from hospital charts and pathological records, and 3% were gathered from subjects' hospital visits.
**Nicaragua (Caldera et al., 2007, 2004)**	Registration of all hospital-admitted suicide attempt cases was conducted at the Hospital Escuela Oscar Danilo Rosales Arguello (HEODRA) hospital in Leon over a three-year period. One nurse took responsibility for the registration system and performed all interviews.
**Pakistan (Shahid et al, 2009)**	A retrospective review of 98 self-harm patients presenting to the emergency department of a tertiary-care teaching hospital in Karachi was carried out. The information recorded included method of self-harm, type, route and quantity of drugs (if used), reason for self-harm, past history, and outcome.
**Panama (González, 2014)**	Since 2005, data on suicide attempters have been collected presenting to the emergency room of the regional hospital Nicolás Alejo Solano de La Chorrera. The information is entered into a paper form by psychiatrists.
**Sri Lanka (Senarathna et al., 2012)**	A study of self-poisoning admissions to all emergency rooms in the Anuradhapura district was carried out from 2008 to 2010.
**Sweden (Tidemalm et al., 2014)**	Data on hospital discharge, cause of death, population and housing census, and education and migration were linked for all people living in Sweden during 1973–1982.
**USA (Barlow et al., 2012)**	With Johns Hopkins University, data on suicide, non-suicidal self-injury, and substance use were analysed from the White Mountain Apache tribally mandated self-injury surveillance registry from 2007 to 2010.

References for examples of individual studies

Barlow A, Tingey L, Cwik M, Goklish N, Larzelere-Hinton F, Lee A, et al. Understanding the relationship between substance use and self-injury in American Indian Youth. Am J Drug Alcohol Ab. 2012;38(5):403–8.

Caldera T, Herrera A, Renberg ES, Kullgren G. Suicide intent among parasuicide patients in Nicaragua: a surveillance and follow-up study. Arch Suicide Res. 2007;11(4):351–60.

Chen VC, Cheng AT, Tan HK, Chen CY, Chen TH, Stewart R, Prince M. A community-based study of case fatality proportion among those who carry out suicide acts. Soc Psychiatr Epidem. 2009;44(12):1005–11.

De Munck S, Portzky G, Van Heeringen K. Epidemiological trends in attempted suicide in adolescents and young adults between 1996–2004. Crisis. 2009;30(3):115–9.

González G. Acerca de la conducta suicida en Panamá (About suicidal behaviour in Panama). Revista De la Sociedad Panameña de Psiquiatría. 2014;2(1):9-12.

Gururaj G, Isaac MK. Epidemiology of suicides in Bangalore. 2001. National Institute of Mental Health and Neurosciences. Bangalore: Vasu.

Medica Mondiale. Dying to be heard – Self-immolation Research Report 2006–2007. Cologne: Medica Mondiale; 2008. (http://www.medicamondiale.org/fileadmin/redaktion/5_Service/Mediathek/Dokumente/English/Documentations_studies/Afghanistan_Dying_to_be_heard_self_immolation_medica_mondiale_2007.pdf, accessed 18 January 2016)

Milner A, Kõlves K, Gladman B, De Leo D. Treatment priority for suicide ideation and behaviours at an Australian emergency department. World J Psychiatr. 2013;3: 34–40.

Peiris-John R, Kafoa B, Wainiqolo I, Reddy RK, McCaig E, Ameratunga SN. Population-based characteristics of fatal and hospital admissions for poisoning in Fiji: TRIP Project-11. Inj Prev. 2013;19(5):355–7.

Rajendra R, Krishna M, Majgi S, Heggere N, Robinson C, Poole R. A feasibility study to establish a Deliberate Self-harm Register in a state hospital in southern India. BJMP. 2015;8(1):a807.

Senarathna L, Jayamanna SF, Kelly PJ, Buckley NA, Dibley MJ, Dawson, AH. Changing epidemiologic patterns of deliberate self-poisoning in a rural district of Sri Lanka. BMC Public Health. 2012;12:593.

Shahid M, Khan MM, Saleem-Khan M, Jamal Y, Badshah A, Rehmani R. Deliberate self-harm in the emergency department: experience from Karachi, Pakistan. Crisis. 2009;30: 85–9.

Sharif-Alhoseini M, Rasouli MR, Saadat S, Haddadi M, Gooya MM, Afsari M, Rahimi-Movaghar V. Suicide attempts and suicide in Iran: Results of national hospital surveillance data. Public Health. 2012;126:990-992.

Sinniah A, Maniam T, Oei TP, Subramaniam P. Suicide attempts in Malaysia from the year 1969 to 2011. Scientific World Journal. 2014;2014:1-13. doi.org/10.1155/2014/718367.

Tidemalm D, Haglund A, Karanti A, Landén M, Runeson B. Attempted suicide in bipolar disorder: risk factors in a cohort of 6086 patients. PLoS One. 2007;9(4):1–9.

Xu Y, Phillips MR, Wang L, Chen Q, Li C, Wu X. Retrospective identification of episodes of deliberate self-harm from emergency room registers in general hospitals: an example from Shanghai. Arch Suicide Res. 2013;17:345–59.
